# Advantages and limitations of experimental autoimmune encephalomyelitis in breaking down the role of the gut microbiome in multiple sclerosis

**DOI:** 10.3389/fnmol.2022.1019877

**Published:** 2022-11-04

**Authors:** Esther Melamed, Jamie L. Palmer, Cara Fonken

**Affiliations:** Department of Neurology, Dell Medical School, University of Texas at Austin, Austin, TX, United States

**Keywords:** EAE, MS, gut microbiome, antibiotics, probiotics, FMT, alcohol, diet

## Abstract

Since the first model of experimental autoimmune encephalomyelitis (EAE) was introduced almost a century ago, there has been an ongoing scientific debate about the risks and benefits of using EAE as a model of multiple sclerosis (MS). While there are notable limitations of translating EAE studies directly to human patients, EAE continues to be the most widely used model of MS, and EAE studies have contributed to multiple key breakthroughs in our understanding of MS pathogenesis and discovery of MS therapeutics. In addition, insights from EAE have led to a better understanding of modifiable environmental factors that can influence MS initiation and progression. In this review, we discuss how MS patient and EAE studies compare in our learning about the role of gut microbiome, diet, alcohol, probiotics, antibiotics, and fecal microbiome transplant in neuroinflammation. Ultimately, the combination of rigorous EAE animal studies, novel bioinformatic approaches, use of human cell lines, and implementation of well-powered, age- and sex-matched randomized controlled MS patient trials will be essential for improving MS patient outcomes and developing novel MS therapeutics to prevent and revert MS disease progression.

## Introduction

Multiple sclerosis (MS) is a chronic autoimmune neurological disease that results in demyelination of the central nervous system (CNS), with symptoms ranging from motor and sensory dysfunction to visual and cognitive deficits, among others (Smith and McDonald, 1999). MS primarily affects individuals in their 20’s and 30’s (Dunn et al., 2015) and is the leading cause of neurological disability in young adults (Koch-Henriksen and Sørensen, 2010). Interestingly, although MS is more common in Northern hemispheres, multiple studies have revealed that the disease affects women more commonly than men across different countries (Ahlgren et al., 2011; Harbo et al., 2013; Bargagli et al., 2016; Almasi-Hashiani et al., 2020).

The incidence of MS has continued to dramatically increase over the past decade, especially in developed countries (Larsen et al., 1984; Svenningsson et al., 1990; Barnett et al., 2003; Koch-Henriksen et al., 2018; Wallin et al., 2019; Walton et al., 2020). Since genetic factors only account for approximately 30% of MS risk, various environmental factors have emerged as potential contributors to MS pathogenesis (Ascherio and Munger, 2008; Alfredsson and Olsson, 2019). Commonly associated environmental factors include latitude, vitamin D deficiency, Westernized diet, gut microbiome dysbiosis, and viral infections, such as Epstein-Barr infection (Alfredsson and Olsson, 2019; Zarghami et al., 2021). It has also been noted that individuals moving from one country to another acquire the risk of MS of the new place of residence, suggesting that environmental factors play an important role in MS disease pathogenesis (Gouider et al., 2022).

Experimental autoimmune encephalomyelitis (EAE) is the most widely used animal model in the development and testing of MS immune therapies and in studying MS disease pathogenesis. Indeed, most of the FDA-approved MS disease-modifying therapies (DMTs) including interferon beta, glatiramer acetate (Teitelbaum et al., 1996), natalizumab (Yednock et al., 1992; Steinman, 2005), sphingosine 1-phosphate modulators (Fujino et al., 2003; Tsai et al., 2016), dimethyl fumarate (DMF) (Schilling et al., 2006), and B cell depletion therapies (Matsushita et al., 2008; Weber et al., 2010) have demonstrated benefits in EAE.

Although EAE is the closest model that approximates human MS, there are important limitations of this model. For example, rodent EAE is primarily confined to the spinal cord with fewer brain lesions (Ransohoff, 2012). In comparison, MS patients develop lesions either in the spinal cord and/or in the brain (Nijeholt et al., 1998). Further, rodent size precludes easy administration of IV preparations of DMTs, evaluation of longitudinal brain volume and cognitive changes, which are all important aspects of human MS. Rodent immune system is also not identical to humans, with differences both in the innate and adaptive immune system (Mestas and Hughes, 2004), potentially limiting some of the immune finding translatability. Side effects of DMTs, such as progressive multifocal leukoencephalopathy (PML), may not be as apparent in EAE animal models (Rudick et al., 2013), making it challenging to design mitigation strategies for DMT side effects without direct human testing. As a result, biological differences between rodents and humans have been implicated in the failure of some of the MS therapeutics in humans, despite initial success in EAE (Weiner et al., 1993).

**FIGURE 1:**
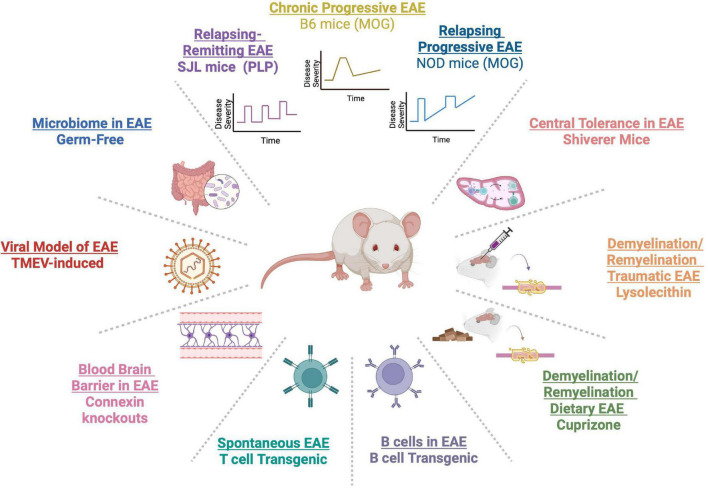
A myriad of experimental autoimmune encephalomyelitis (EAE) models have been developed to capture varied aspects of the clinical course of multiple sclerosis (MS) [C57BL/6 (B6), SJL/J, and PL/J mice]. Genetic models have allowed the study of T and B cells in MS in TCR and B-cell receptor (BCR) transgenics. Germ-free models have enabled the study of the gut microbiome. Viral models, such as Theiler’s murine encephalomyelitis virus (TMEV), have elucidated viral contribution to MS. MS-related demyelination and remyelination has been studied in cuprizone and lysolecithin models. Connexin knockouts have been used to study the role of the blood brain barrier (BBB) in neuroinflammation. Central tolerance has been examined in shiverer transgenic mice.

Given the diverse risk factors for MS, variation in disease presentation and clinical course, different involved immune cell types, and genetic and environmental risk factors, there is not one EAE animal model that can entirely recapitulate all of MS phenotypes. To best capture vital aspects of MS, multiple EAE models have been developed (Figure 1). For example, EAE induction with myelin oligodendrocyte glycoprotein (MOG) in C57BL/6 (B6) mice, has been utilized to study chronic disease (Amor et al., 1994; de Rosbo et al., 1995). Induction of SJL/J mice with proteolipid protein (PLP_139–151_), leads to a relapsing remitting EAE course (McRae et al., 1992), approximating relapsing remitting MS (RRMS). In PL/J mice, immunization with MOG, induces a chronic relapsing EAE course, similar to progressive relapsing MS (Amor et al., 1994). Use of non-obese diabetic mice (NOD) mice immunized with MOG can emulate secondary progressive MS (SPMS) course (Degenhardt et al., 2009; Simmons et al., 2013). Studies of *shiverer* mice, with deletion of myelin basic protein (MBP) exons 7–11, have helped to investigate the role of central tolerance in MS (Harrington et al., 1998). B cell deficient mice have helped to learn about the contribution of B cells in MS pathophysiology (Smith et al., 2005). Spontaneous EAE, which emulates genetic predisposition in MS, has been best studied in T-cell receptor (TCR) transgenic mice specific for MOG_92–106_ (2D2) (Goverman et al., 1993; Brabb et al., 1997; Bettelli et al., 2003). Of note, addition of MOG-specific B cells by crossing 2D2 mice to MOG-specific Ig heavy chain knock-in mice can increase spontaneous EAE up to 60% (Bettelli et al., 2006).

Several models have also been developed to understand the process of demyelination and remyelination in MS. These models include the use of dietary cuprizone, a copper-chelating agent that leads to demyelination (Hiremath et al., 1998; Matsushima and Morell, 2001), whereby cessation of dietary cuprizone prompts remyelination. In addition, surgical model of focal demyelination with lysolecithin can be a powerful way to examine local demyelination in context of traumatic injury (Keough et al., 2015). Further, given the importance of viral infections in MS (Marrodan et al., 2019), Theiler’s murine encephalomyelitis virus (TMEV) models have been used to study how preceding viral infections can model MS-related neuroinflammation (McCarthy et al., 2012). The use of germ-free (GF) animals has allowed to examine the role of gut microbiome in MS (Berer et al., 2011; Lee et al., 2011). Also, researchers have used connexin 32 or 47 knockout mice in EAE to study the role of the blood brain barrier (BBB) in MS pathogenesis (Stavropoulos et al., 2021). The availability of multiple EAE models has allowed access to mechanistic studies that can inform human MS pathophysiology.

In addition to lessons gleaned from EAE studies about MS pathophysiology and DMTs, EAE has also been a useful tool to learn about modifiable environmental MS factors and discover opportunities for therapeutics based on these factors. In this review, we will discuss how MS and EAE studies compare in our understanding of environmental factors in MS, focusing on the gut microbiome, dietary factors, alcohol, probiotics, and antibiotics, as well as potential risks and benefits of fecal microbiome transplants (FMT).

## Gut microbiome

The gut microbiome is composed of trillions of bacteria, viruses, and fungi whose collective genes outnumber human genes by 150 times (Qin et al., 2010; Ochoa-Repáraz et al., 2018). Not only does the gut microbiome help to maintain gastrointestinal (GI) homeostasis, but the microbiota have important influences on the rest of the body and the brain (Dantzer et al., 2008; Duerkop et al., 2009; Forsythe and Bienenstock, 2010; Cryan and Dinan, 2012). For example, there is bidirectional communication between the gut and the central nervous system (CNS), with the microbiota considered to be essential for the development of the immune system and playing a major role in influencing the nervous system (Cryan and O’Mahony, 2011; Bhuiyan et al., 2021) *via* bacterial metabolites (Kaur et al., 2019), neurotransmitters (Barrett et al., 2012; Erny et al., 2015) and short-chain fatty acids (SCFAs) (Hoyles et al., 2018; Spichak et al., 2021). In turn, the CNS has a critical role in modulating gut-related metabolism and physiology *via* the vagus nerve (Wang et al., 2002; Miyauchi et al., 2022).

Alterations in the gut microbiome have been widely documented both in patients with MS and animals with EAE. Specifically, multiple studies have now established dynamic alterations in the gut microbiome taxa at different stages of MS and in response to treatment with different DMTs. In turn, EAE studies have contributed to our evolving understanding of mechanisms behind microbiota influences in MS. Potential therapeutic interventions targeting the gut microbiome in MS and EAE, such as dietary interventions, probiotics, antibiotics, SCFAs, and FMT may offer an exciting avenue for treating MS in addition to or in concert with DMTs. In the following sections, we will review the evolving landscape of gut microbiome mechanisms and therapeutics in MS and EAE.

## Gut microbiome studies in multiple sclerosis

Multiple sclerosis patients have been documented to have a decreased biodiversity (Swidsinski et al., 2017; Navarro-López et al., 2022) of microbiome as well as gut dysbiosis (Miyake et al., 2015), or alteration in gut microbiota from homeostatic commensal species. Specifically, there is a reduction in Prevotella (Chen et al., 2016; Berer et al., 2017; Cekanaviciute et al., 2017; Cosorich et al., 2017; Ventura et al., 2019; Duscha et al., 2020; Takewaki et al., 2020), Bacteroidetes, especially in the butyrate producer, *Butyricimonas*, Clostridia clusters IV and XIV (Miyake et al., 2015; Duscha et al., 2020), *Bacteroidaceae* family, *Faecalibacterium* genera (Miyake et al., 2015), and *Ruminococcus genera* (Takewaki et al., 2020). Several studies have also documented an overrepresentation of gut bacteria such as Streptococcus (Miyake et al., 2015; Cosorich et al., 2017; Takewaki et al., 2020), *Methanobrevibacter smithii* (Jangi et al., 2016), and *Akkermansia* genera (Jangi et al., 2016; Berer et al., 2017; Cekanaviciute et al., 2017; Ventura et al., 2019; Duscha et al., 2020; Takewaki et al., 2020) compared to healthy controls. In pediatric MS patients, increased levels of *Clostridium, Bilophila, Escherichia*, and Shigella have been found along with a decrease in the levels of *Eubacterium rectale* and *Corynebacterium* (Tremlett et al., 2016). Interestingly, absence of *Fusobacteria* in these patients corresponded to a shorter time of relapse (Tremlett et al., 2016).

MS studies have also suggested that DMTs can affect the gut microbiome environment. For example, Jangi et al. (2016) found that Prevotella and Sutterella increased with interferon beta and glatiramer acetate treatment while *Sarcina* decreased compared to untreated MS patients. In another study, Lachnospiraceae and Veillonellaceae families decreased in MS patients in response to glatiramer acetate and DMF (Katz Sand et al., 2019). DMF was further associated with a decrease in phyla Firmicutes and Fusobacteria, and the order Clostridiales as well as an increase in phylum Bacteroidetes (Katz Sand et al., 2019).

There are several important limitations to human microbiome studies that necessitate complementary evaluation in animal models. For example, human studies on the microbiome have been primarily limited to analysis of fecal samples despite known variations in microbiota composition along the GI tract. Specifically, there are geographical differences between the small intestine, where most of the nutrient absorption occurs, and the large intestine, which is responsible for fecal concentration and disposal. Accordingly, these regions host different gut microbiota. Most human studies have focused on fecal samples, largely due to the difficulty in getting samples from other parts of the GI tract. However, without adequate sampling of different parts of the gut and understanding dynamics of the gut microbiome across the GI tract, conclusion based primarily on fecal microbiota may not fully recapitulate gut microbiome dynamics in MS. In addition, it is challenging to study the gut microbiome in humans, due to varying diets and intermittent use of antibiotics and other medications that can shift the gut microbiome as well as individuals traversing geographic lines and getting exposed to different environmental factors.

## Gut microbiome studies in experimental autoimmune encephalomyelitis

Given the limitations of human studies, it has been critical to use animal models to better understand the role of the gut microbiome in MS autoimmunity. Studies in EAE have intriguingly demonstrated that the gut microbiome is imperative to the onset of autoimmunity. To this end, GF animals do not develop EAE and require transfer of gut microbiome either from donor animals with EAE or from patients with MS (Mackie et al., 1999; Berer et al., 2017; Cekanaviciute et al., 2017). Using EAE models, it has been observed that autoreactive MOG-specific T cells are abundant in the gut during EAE, suggesting that these T cells could be interacting with gut bacteria prior to their egress to the CNS (Duc et al., 2019; Miyauchi et al., 2020). Further, administration of specific microbiota such as *Lactobacillus murinus* has led to disease amelioration in EAE (Wilck et al., 2017). In addition transfer of *Erysipelotrichaceae* and *Limosilactobacillus reuteri* (*L. reuteri*) can result in more severe EAE symptoms compared to mono-colonization with *L. reuteri* (Miyauchi et al., 2020). Other studies have revealed that genetic background can interact with the gut microbiome. For example, in a study of MS twins, with one healthy twin and the other twin with MS, transfer of gut microbes from the MS twin led to higher EAE incidence and reduction in interleukin (IL)-10 compared to transfer from the healthy twin (Berer et al., 2011, 2017; Cekanaviciute et al., 2017).

Other EAE studies have clarified potential mechanisms of how gut microbiota may influence the nervous system. For example, SCFAs, such as butyrate, propionate and acetate, can modulate regulatory T cells (T regs) in the gut and their supplementation can lead to EAE amelioration (Haghikia et al., 2015; Duscha et al., 2020; Silva et al., 2020; Trend et al., 2021). Butyrate has specifically been shown to modulate remyelination, and therefore, use of SCFAs offers a promising avenue for affecting long term neurological recovery (Chen et al., 2019). In addition, microbiota can shape the integrity of the gut-blood and BBB (Parker et al., 2020). For example, GF mice have a greater permeability in the BBB and gut-blood barrier compared to animals reared in specific pathogen free (SPF) conditions (Cryan et al., 2020). Transfer of gut bacteria, such as *Clostridium tyrobutyricum*, or oral supplementation with the SCFA butyrate, has been linked with stabilization of the BBB by upregulating tight junction proteins (Braniste et al., 2014). Another potential source of neuroprotective gut microbial products in EAE are bacterial metabolites (Elsayed et al., 2022). For example, gut bacteria, such as Lactobacillus, are able to metabolize tyrosine, phenylalanine, and tryptophan into metabolites that cross the BBB and impact reactive oxygen species and downstream neuroinflammation *via* the aryl hydrogen receptor (AHR) (Kaur et al., 2019). In addition, bacterial metabolites such as phytoestrogens can lead to EAE amelioration by decreasing inflammatory cytokines and modulating leukocyte trafficking of into the CNS (De Paula et al., 2008).

Furthermore, EAE studies have revealed that several gut bacteria can influence the immune system. For example, microbiota can influence populations of Tregs, Bregs, and IgA+ plasma cell populations (Ochoa-Repáraz et al., 2010a; Opazo et al., 2018; Pröbstel et al., 2020; Boussamet et al., 2022). Specific microbiota, such as Erysipelotrichaceae and segmented filamentous bacteria, have the ability to stimulate T helper (Th) 17 responses (Cryan et al., 2020; Miyauchi et al., 2020), including production of *Il17a, Csf2* [encoding granulocyte macrophage colony-stimulating factor (GM-CSF) and *Il23r*], which are known to be inflammatory in EAE and MS. On the other hand, *Prevotella* has been found to induce Tregs in mesenteric lymph nodes and the spleen and to suppress Th1 and Th17 cells in EAE mice (Mangalam et al., 2017). Likewise, other EAE studies have revealed that gut bacteria components, such as polysaccharide A (PSA) from *Bacteroides fragilis* are immunoprotective (Erturk-Hasdemir et al., 2021). For example, PSA was shown to induce accumulation of gut-derived Treg cells and promote expansion of CD39 + CD4 + Treg cells, which migrate to the CNS (Ochoa-Repáraz and Kasper, 2017). Treatment with PSA prophylactically or during the course of EAE is sufficient to prevent EAE in an IL-10-dependent manner (Ochoa-Repáraz et al., 2010b; Liu et al., 2019; Gabanyi et al., 2022).

### Antibiotics

Antibiotics are critical medications that have revolutionized medicine and have helped to save millions of lives by treating bacterial infections. Intriguingly, antibiotics also have the power to affect autoimmunity (Strzępa et al., 2018). In addition to their direct effects of eradicating specific types of bacteria, antibiotics also modulate the immune system by inhibiting T cell proliferation, inflammatory cytokine and chemokine production, and phagocytosis (Singh et al., 2021). Moreover, antibiotics alter gut flora by virtue of inhibiting growth of certain bacteria, which can destabilize gut microbiota networks, allowing some bacteria to become more abundant. An important example of this synergistic network behavior is overpopulation of *Clostridium difficile* in patients on longstanding antibiotics, which can lead to a diarrheal illness in humans, and in some cases may require a microbiome transfer to reverse (Rao and Safdar, 2016). At the same time, the right balance of affecting microbial networks in the gut can have the great potential of alleviating or preventing autoimmunity by modulating pathogenic and beneficial gut bacterial taxa (Strzępa et al., 2018).

## Antibiotic studies in multiple sclerosis

In human MS studies, there have been mixed results in terms of association of antibiotics with amelioration vs. worsening of MS (Table 1). Metz et al. (2017) evaluated a cohort of 142 patients, 68% females and 32% males, with CIS and demonstrated a lower incidence of clinically definite MS in the minocycline treated group (*n* = 72) during 6 months but not during 24 months. Of note, only 17 minocycline-treated and 12 control-treated patients were evaluated at 24 months, lowering power for the 24-month analysis. More participants in the minocycline group experienced adverse events, such as rash, tooth discoloration, and dizziness. In a smaller study, 15 patients with RRMS on interferon therapy, were treated with doxycycline for 4 months and experienced improvement in EDSS and contrast-enhancing MRI lesions without experiencing any adverse side effects (Minagar et al., 2008). In a similar study design, 60 RRMS and SPMS patients with breakthrough disease, consisting of 88% female and 12% males, were treated with a combination of interferon and doxycycline therapy for 6 months (Mazdeh and Mobaien, 2012). In this cohort, patients experienced an improvement in clinical EDSS scores and 13% of patients experienced radiological improvement, while 15% demonstrated radiological worsening in terms of contrast enhancing lesions. The human study results suggest that there may be an interaction between the type of MS (i.e., CIS vs. RRMS vs. SPMS) and type of antibiotic administration that may contribute to neuroinflammatory outcomes in response to antibiotic therapy.

**TABLE 1:** Effects of antibiotic administration on multiple sclerosis (MS) risk and progression.

	# of patients	Sex/Age	Type of MS	Antibiotics used	Duration of treatment	Effect on MS	References
Protective	*n* = 142 (72 receiving antibiotics)	18–60 years (mean = 35.8) 68.3% female, 31.7% male	CIS	100 mg minocycline twice/day (oral)	24 months or until the time of MS diagnosis	Reduced clinical conversion rates to MS	Metz et al., 2017
	*n* = 15	19–57 years (mean = 44.5); 80% female, 20% male	RRMS	100 mg doxycycline daily (oral; in combination with IFN-β1a)	4 months	Reduction in Gd + lesions, decreased EDSS score	Minagar et al., 2008
	*n* = 60	14–51 years (mean = 32); 88.3% female, 11.7% male	RRMS, SPMS	100 mg doxycycline daily (in combination w/IFN-β1a)	6 months	Decreased EDSS scores, decreased relapse rate	Mazdeh and Mobaien, 2012
	*n* = 163	Mean = 36.2 years; Sex breakdown unavailable	RRMS, SPMS, PPMS	Anti-*Chlamydophila* antibiotics, penicillins, cephalosporins, tetracyclines, macrolides, other (unlisted)	Range of durations	>2 weaks penicillin and >1 week tetracycline reduced risk of MS	Alonso et al., 2006
Detrimental	*n* = 3,259	Lists age range categories from <30 to ≥50; 67% female, 33% male	Unavailable	Pivmecillinam, macrolides, tetracyclines, sulfonamides/trimethroprim, nitrofurantoin, quinolones, metronidazole, penicillin	Range of durations	Penicillin and other antibiotics taken within the year before clinical MS onset was associated with increased MS risk	Nørgaard et al., 2011

CIS, clinically isolated syndrome; RRMS, relapsing-remitting MS; Gd+, gadolinium-enhancing; EDSS, Expanded Disability Status Scale; SPMS, secondary progressive MS; PPMS, primary progressive MS; IFN, interferon.

Two retrospective cohort studies have examined association between prior ever antibiotic use and MS incidence. In one study, 163 patients with RRMS were evaluated in a British database for antibiotic use in the prior 3 years (Alonso et al., 2006). Compared to other antibiotics, tetracycline use for more than 1 week and penicillin for 2 weeks was associated with a decreased MS risk. In a nationwide case-control study in Denmark, authors evaluated data for 3,259 patients with MS (2/3 women and 1/3 men) and 32,590 case controls on whether prior antibiotic use was associated with MS diagnosis. In this study, it was found that prior use of penicillin and other antibiotics was associated with increased risk of MS diagnosis. The implication from these retrospective studies is that prior infections necessitating use of antibiotics may have a link to MS (Nørgaard et al., 2011).

## Antibiotic studies in experimental autoimmune encephalomyelitis

Several studies in different EAE models have provided evidence for protective effects of antibiotics (Table 2). For example, oral treatment with antibiotics in a study of female SJL and B6 mice treated with ampicillin, neomycin, metronidazole and vancomycin for 1 week prior to EAE induction, resulted in disease amelioration in both strains along with increased protective cytokines, IL-10 and IL-13, and enhanced frequency of Tregs (Ochoa-Reparaz et al., 2009). In another study, treatment with the same cocktail of antibiotics either orally or *via* intraperitoneal (IP) route in B6 mice 1 week prior to EAE induction, demonstrated EAE amelioration in the group that received oral antibiotics compared to the IP route. EAE-protected animals had higher CD19 + B220+ and CD19 + CD5+ B cell cells in the lymphoid organs (Ochoa-Repáraz et al., 2010a). The results of this study suggest that IP antibiotics may not be as effective in EAE amelioration due to bypassing the gut. Similarly, Yokote et al. (2008) found that B6 female mice treated with a mixture of non-absorbing oral antibiotics kanamycin, colistin, and vancomycin orally for 1 week prior to EAE induction led to EAE amelioration, lower frequency of infiltrating T cells in the CNS, decreased demyelination in the lumbar spine, lower production of Th17-related cytokines in the intestinal lamina propria, as well as invariant natural killer T (NKT) cell depletion in association with alteration in gut bacteria.

**TABLE 2:** Effects of antibiotic administration on experimental autoimmune encephalomyelitis (EAE) development/progression and myelination.

	# of animals	Sex/Age	Strain	Type of EAE induction	Antibiotics used/Route of administration	Duration of antibiotic administration	EAE/Myelination outcomes	References
Protective	*n* = 12/group (no treatment, IP, oral; for SJLs, 12 mice for drinking water and 12 mice for oral gavage)	Females; 6 weeks	SJL and C57BL/6 mice	Active induction; PLP_139–151_ (SJL) and MOG_35–55_ (C57BL/6)	Ampicillin (1 g/mL) Metronidazole (1 g/mL) Neomycin sulfate (1 g/mL) Vancomycin (0.5 g/mL) IP or oral supplementation through drinking water; oral gavage also used for SJLs	1 week leading up to induction	Reduced EAE scores (oral administrations only)	Ochoa-Reparaz et al., 2009
	*n* = 10/group (PBS-treated/oral/IP)	Sex/age unavailable	C57BL/6 mice	Active induction (MOG_35–55_)	Ampicillin (1 g/mL) Metronidazole (1 g/mL) Neomycin sulfate (1 g/mL) Vancomycin (1 g/mL) IP injections or oral supplementation through drinking water	1 week leading up to induction	Reduced EAE scores (oral administration only)	Ochoa-Repáraz et al., 2010a
	*n* = 5/group (ABX or no ABX)	Female; 6 weeks	C57BL/6 mice	Active induction (MOG_35–55_)	Colistin (2000 U/mL) Kanamycin (1 mg/mL) Vancomycin (0.1 mg/mL) Oral supplementation through drinking water	1 week prior to EAE induction, continued for duration of experiment	Reduced EAE scores; reduced demyelination/cellular infiltration	Yokote et al., 2008
	*n* = 8–12/group	Female; 8–10 weeks	C57BL/6 mice	Active induction; MOG_35–55_	Ampicillin (1 g/L) Metronidazole (1 g/L) Neomycin sulfate (1 g/L) Vancomycin (0.5 g/L) Oral gavage	3 days leading up to induction	Prophylactic treatment reduced EAE scores	Seifert et al., 2018
	*n* = 8/group (ABX admin day of induction, day of symptom onset, and control)	Female; 6–8 weeks	C57BL/6 mice	Active induction; MOG_35–55_	Ceftriaxone (200 mg/kg/day) IP injection	ABX started either day of induction or day of symptom onset, continued until end of experiment	Both ABX administration times reduced EAE scores	Melzer et al., 2008
	*n* = 6–12/group (PBS, antibiotics, WT *B. fragilis* recolonized, ΔPSA *B. fragilis* recolonized)	Female; 6 weeks	SJL mice	Active induction; PLP_139–151_	Ampicillin (1 g/mL) Metronidazole (1 g/mL) Neomycin sulfate (1 g/mL) Vancomycin (0.5 g/mL) Oral supplementation through drinking water	1 week of ABX, then 1 day bacterial reconstitution or sham, followed by induction 1 week later	Reduced EAE scores	Ochoa-Repáraz et al., 2010b
	*n* = 5–10/group (ABX-ABX, ABX-vehicle, ABX-probiotics, vehicle alone, sham mice given ABX)	Female; TMEV inoculation at 4 weeks, ABX started 55 days after	SJL mice	TMEV infection	Ampicillin (1 g/L) Metronidazole (1 g/L) Neomycin sulfate (1 g/L) Vancomycin (0.5 g/L) Oral supplementation through drinking water	15 or 30 days	Improved motor function; lesser cellular infiltration	Mestre et al., 2019
Detrimental	*N* = 4–6/group (see Figure 1)	Female; 4 months	C57BL/6 mice	Lysolecithin-induced lesions	Ampicillin/sulbactam (1.5 g/L) Ciprofloxacin (200 mg/L) Imipenem (250 mg/L) Metronidazole (1 g/L) Vancomycin (500 mg/L) Oral supplementation through drinking water	8 weeks prior to lesioning, continued for duration of experiment	Limited OPC differentiation and efficient removal of myelin debris by 14 days post-lesioning	McMurran et al., 2019
	*N* = 15/group (control, azithromycin, clarithromycin)	Female; 7 weeks	Lewis rats	Active induction; guinea pig MBP_68–86_	Azithromycin (50 mg/100 g body weight) Clarithromycin (50 mg/100 g body weight) Oral gavage	One administration 2 days prior to induction	Exacerbated EAE scores; reduced iNOS mRNA expression in SC	Wang et al., 2009
	*N* = 15/group (ABX, control)	Pregnant females given ABX (age unavailable); offspring induced w/EAE at 8 weeks (sex unavailable)	Dark-agouti rats	Rat SCH	Neosulfox (Sulfadimidine sodium 10% (w/w), neomycin sulfate 6%, oxytetracycline hydrochloride 4%) 2.5 g/l Pentrexyl (ampicillin) Total max dosage of 2.5 g/L Oral supplementation through drinking water	Dams started on ABX 2 weeks before giving birth, continued until 4 weeks post-birth	Early life administration exacerbated EAE scores; more immune infiltration into SC	Stanisavljević et al., 2019
No effect/mixed	*N* = 10/group (ABX/control)	2 weeks or 4 weeks post-birth; Sex unavailable	OSE mice	Spontaneous model	Ampicillin (1 g/L) Neomycin (1 g/L) Vancomycin (1 g/L) Oral supplementation through drinking water	2 weeks	Prophylactic ABX treatment reduced EAE scores Therapeutic administration of ABX did not significantly affect demyelination in spinal cord or optic nerve	Gödel et al., 2020
	*N* = 10/group (non-EAE + vehicle, non-EAE + ABX, EAE + vehicle, EAE + ABX)	Female; PD21-PD105 (EAE induced on PD80)	C57BL/6	Active induction; MOG_35–55_	Ampicillin (1 mg/mL) Metronidazole (10 mg/mL) Neomycin (10 mg/mL) Vancomycin (5 mg/mL) Antifungal Amphotericin-B (0.1 mg/mL) Oral supplementation through drinking water	10–12 weeks	Delayed EAE onset; higher severity from days 19–22 post-induction, reduction of symptoms from day 24–25	Zeraati et al., 2019

IP, intraperitoneal; PLP, proteolipid protein; MOG, myelin oligodendrocyte protein; PBS, phosphate buffered solution; OPC, oligodendrocyte progenitor cell(s); MBP, myelin basic protein; iNOS, inducible nitric oxide synthase; SC, spinal cord; ABX, antibiotic(s); PSA, polysaccharide A; TMEV, Theiler’s murine encephalomyelitis virus; SCH, spinal cord homogenate; PD, postnatal day.

Similar findings have been described in other EAE models. Mestre et al. (2019) administered ampicillin, metronidazole, neomycin, and vancomycin during the pre-symptomatic stage to female SJL/J mice infected with TMEV and compared three treatment groups who either continued on antibiotics for 30 days, stopped antibiotics after 15 days, or were given probiotics after 15 days. Interestingly, antibiotic-treated mice did not develop motor dysfunction, had fewer infiltrating CD4^+^ and CD8^+^ T cells in the CNS, and had ameboid shape of microglia, suggesting an anti-inflammatory state, compared to the probiotic treated group that experienced worse motor function and axonal integrity (Mestre et al., 2019). In a lysolecithin EAE model, McMurran et al. (2019) treated female B6 mice with ampicillin/sulbactam, ciprofloxacin, vancomycin, metronidazole, and imipenem for 8 weeks followed by lysolecithin injection. It was found that the antibiotic-treated animals experienced a decrease in demyelination with an associated decrease in microglia in lesions, though oligodendrocyte progenitor cell (OPC) differentiation and remyelination was not affected. Of note, the animals in this experiment were older (4–7 months old) than in many of the other antibiotic treatment groups (6 weeks old). In a spontaneous EAE model, Gödel et al. (2020) used oral antibiotic treatment in opticospinal (OSE, 2D2 × IgHMOG C57BL/6) mice at 2–4 weeks of age and either continued or discontinued antibiotics for an additional 2 weeks following symptom initiation. Their results demonstrate that antibiotic treatment led to prevention of spontaneous EAE in association with decreased gut microbiota alpha diversity and an increase in species within the genera *Akkermansia, Bacteroides*, and *Blautia*. The authors also found that antibiotic treatment of affected OSE mice did not affect ongoing CNS autoimmune disease, suggesting that timing of antibiotic administration pre-EAE may be more beneficial in modulating the gut microbiome and CNS disease compared to post-disease treatment.

Of note, there can also be potential aggravating antibiotic side effects based on animal studies (Table 2). For example, in a neonatal study of DA rats (Stanisavljević et al., 2019), administration of oral Neosulfox, neomycin, oxytetracycline, and ampicillin to pregnant dams followed by 4 weeks of treatment postnatally prior to EAE induction led to disease exacerbation in antibiotic treated animals compared to controls. The results of this study suggest that the type of antibiotic mix and prenatal/postnatal timing of antibiotic intake may negatively impact neuroinflammation. In another study, animals treated with antibiotics and anti-fungal agents (neomycin, ampicillin, metronidazole, vancomycin, and amphotericin) from early adolescence [postnatal day (PD) 21 until adulthood (PD 105)] displayed a delay in EAE onset with corresponding decrease in IFN-γ and IL-17A levels and increased IL-10. These animals were also found to have higher levels of anxiety and depression compared to the non-antibiotic treated group (Zeraati et al., 2019). Notably, the animals in this study were treated for the longest duration compared to other studies, suggesting that the length of time of antibiotic treatment may contribute to psychiatric symptoms.

In summary, while most studies agree on the protective effects of antibiotics in EAE and MS, it should be noted that antibiotic administration, especially with broad spectrum antibiotics, could have unwelcome consequences, such as eradication of commensal species, GI upset with diarrheal illness, and a potential increase in anxiety. Further studies in animal models will need to be performed to clarify the timing, type and dose of antibiotics that may be protective vs. detrimental, as well as how males versus females at different ages and experiencing different types and stages of MS may respond to antibiotic treatment in terms of demyelination and remyelination. In addition, further studies in patients will also need to compare single vs. combination antibiotic use in ameliorating vs. worsening MS incidence and progression in males and females of different ages. Lastly, as antibiotics can promote anti-inflammatory actions in the body, future studies will need to address mechanistically to what degree antibiotics act *via* the gut microbiome vs. directly on the immune system.

## Potential therapeutic strategies targeting the gut microbiome

### Probiotics

Probiotics are live microorganisms, presumed to be beneficial to the host, that have gained popularity in recent years as supplements to modulate the gut microbiome. Multiple types of probiotics are widely available to consumers and are currently not regulated. For patients with autoimmune conditions, such as MS, the goal is to define a set of microorganisms for the probiotic preparation that would exert long-lasting immunomodulatory effects.

Probiotics have now been studied in both human MS and EAE. In MS studies, positive benefits of different probiotic combinations include improvement in clinical scores such as the Expanded Disability Status Scale (EDSS), Beck Depression Inventory (BDI)-II, and General Health Questionnaire (GHQ), and mental health, correlating with modulation of brain-derived neurotrophic factor (BDNF), IL-6, suppression of IL-17 CD4 + T cells, enhanced Tregs and regulatory B cells (Bregs) and expression of IL-10 (Kouchaki et al., 2017; Salami et al., 2019; Mestre et al., 2020; Jiang et al., 2021; Rahimlou et al., 2022). Similarly, in animal EAE studies, probiotic administration has been demonstrated to delay EAE onset, reduce disease severity and progression *via* maintaining mucosal barrier in the gut (Ohland and MacNaughton, 2010), increase production of immunoglobulin (Ig)As (Liu et al., 2020), as well as enhance abundance of SCFA-producing bacteria while decreasing abundance of pathogenic bacteria (He et al., 2019; Mestre et al., 2020).

## Probiotic studies in multiple sclerosis

Three randomized control trials (RCT) in RRMS patients have been reported in reference to probiotic supplementation. In one double blind RCT (Kouchaki et al., 2017), 30 participants received a combination probiotic containing *Lactobacillus acidophilus, Lactobacillus casei, Lactobacillus fermentum, and Bifidobacterium bifidum* for 12 weeks. The cohort consisted of about 83% females and 16.7% males with patients on interferon therapies, ages 18–55 and healthy controls who were treated with starch containing placebo pills for the duration of the study. In this study, Kouchaki et al. (2017) reported that the probiotic-treated group experienced an improvement in EDSS, BID, depression, anxiety, and stress scale as well as general health questionnaire. In addition, participants on probiotics had lower C-reactive protein (CRP), malondialdehyde and insulin (MDA), and higher plasma nitric oxide (NO). In another double blind RCT, Salami et al. (2019) evaluated 48 RRMS patients on interferon therapy, 20–60 yo, consisting of 75% female and 25% male participants, divided between probiotic and control treatments for 16 weeks. Patients in the probiotic group received a probiotic containing a combination of *Lactobacillus and Bifidobacterium*: *Bifidobacterium infantis, Bifidobacterium lactis, Lactobacillus reuteri, L. casei, Lactobacillus plantarum, and L. fermentum*, while controls were given maltodextrin tablets. Similarly to the Kouchaki et al. (2017) study, the authors reported an improvement in EDSS, depression and general health questionnaires and a decrease in levels of CRP, MDA, and IL-6, and with increased concentration of NO and IL-10. In the longest running RCT, Rahimlou et al. (2022) studied 70 DMT-untreated RRMS patients, 18–50 yo for 6 months, randomized to probiotic (*n* = 35) vs. placebo (*n* = 35). In this study, 72% of participants were female and 28% were male and probiotic treatment consisted of two probiotic pills daily that collectively contained 14 different Bifidobacterium and Lactobacillus bacterial strains. The patients receiving probiotic treatment experienced an improvement in general and mental health and pain scales, including GHQ-28, BDI-II, Fatigue Severity Scale, and Pain Rating Index as well as an increase in BDNF and decrease in IL-6 levels.

## Probiotic studies in experimental autoimmune encephalomyelitis

### Lactobacillus

One of the most widely used probiotics in human and animal studies is the genus *Lactobacillus* (L.), composed of over 260 diverse species that reside in and influence different parts of the body. Many of the Lactobacillus species are also found in animal hosts (Zheng et al., 2020), thus facilitating the study of probiotics in animal models of MS. EAE studies have linked some of the Lactobacillus species as beneficial and others as detrimental. For example, Johanson et al. (2020) found that supplementation with *L. reuteri* in female B6 MOG-induced EAE animals significantly ameliorated EAE signs. Another study in MOG-induced EAE in female B6 mice corroborated these results, demonstrating that treatment with *L. reuteri* led to reduction in EAE scores and increased diversity of gut microbiota in correlation with downregulation of Th1 and Th17 immune responses (He et al., 2019). In a Dark Agouti (DA) female rat model of EAE, γ-aminobutyric acid (GABA)-producer *L. brevis* was shown to improve EAE outcomes in terms of delaying the onset, decreasing the duration and EAE disease severity (Sokovic-Bajic et al., 2020). In a cuprizone EAE model of female B6 mice, *L. casei* attenuated motor impairment during demyelination and shifted Th17 cells to Tregs (Gharehkhani Digehsara et al., 2021).

In several studies, co-administration of different Lactobacillus species or Lactobacillus in combination with other probiotics microorganisms was also found to be protective. For example, supplementation of *L. crispatus, L. rhamnosus*, and *Bifidobacterium animalis* strains in female Lewis rats revealed a delay in MBP-induced EAE onset as well as prevention of spinal cord demyelination in correlation with a higher level of Transforming Growth Factor-β (TGFβ) and Tregs (Consonni et al., 2018). In a comparative study of *B. animalis* and *L. plantarum*, administration of both strains was found to lead to significant delay in MOG-induced EAE progression in female B6 mice relative to supplementation with individual strains (Salehipour et al., 2017). Two studies, one in SJL/J females in a TMEV model of EAE (Mestre et al., 2020) and another study in MOG-induced EAE in B6 female mice (Calvo-Barreiro et al., 2020), reported that treatment with Vivomixx, a mix of eight probiotics from genera *Lactobacillus, Bifidobacterium*, and *Streptococcus*, led to improvements in motor scores, increases Bregs and IL-10, as well as enhanced levels of plasma SCFAs, butyrate and acetate.

Not all EAE studies, however, have agreed regarding beneficial properties of Lactobacillus supplementation. For example, Montgomery et al. (2020) reported that *L. reuteri*, a commensal species in wild type mice can exacerbate EAE in another animal strain. In this study, the authors isolated *L. reuteri* from cecal contents of PWD/PhJ mice, performed a transfer into 4-week-old GF recipient B6 mice, and evaluated EAE in F1 offspring of the recipients. Compared to controls not colonized with *L. reuteri*, GF offspring of *L. reuteri* recipients experienced higher EAE scores as well as higher frequency of GM-CSF and IFN-γ producing T cells in the spinal cord. In analyzing the role of *L. reuteri* in EAE amelioration vs. exacerbation, Miyauchi et al. (2020) studied *L. reuteri* colonization in female GF B6 mice either independently or together with Erysipelotrichaceae bacterium (OTU0002). They observed that mono-colonization with L. reuteri did not affect EAE severity, while co-colonization with OTU0002 led to higher EAE incidence and clinical scores, enhanced demyelination, and cell infiltration in the spinal cord *via* generation of higher Th17 cells and molecular mimicry to MOG. Based on this study, the authors concluded that there is cooperation between different bacterial species to trigger EAE exacerbation. The fact that this synergism drastically changed the effect of *L. reuteri*, indicates the challenges in studying individual probiotic species when complex interactions exist within the gut microbiota networks.

### Prevotella

The genus *Prevotella* in the order *Bacteroidales* is another group of commensal bacteria implicated in immunity. There are 25 species of *Prevotella* that have been noted to have both beneficial and detrimental roles. One of the studied species in MS and EAE is *Prevotella histicola*, found to be decreased in both MS and EAE studies, and proposed as an MS probiotic (Mangalam and Murray, 2019; Shahi et al., 2019; Mandić et al., 2022). Notably, levels of *Prevotella* increase with interferon and glatiramer acetate treatment (Jangi et al., 2016; Cosorich et al., 2017).

*Prevotella histicola* has also been linked with EAE disease suppression in a dose dependent manner in a human leukocyte antigen (HLA)-class II transgenic mouse model of EAE (Shahi et al., 2019). In this study, *P. histicola* supplementation decreased EAE disease severity comparably to glatiramer acetate. However, co-administration of *P. histicola* and glatiramer acetate did not provide additional benefit in EAE outcomes (Shahi et al., 2019). In another study, Mangalam et al. (2017) found that *P. histicola* may be suppressing neuroinflammation *via* modulation of frequency and function of antigen presenting cells and inducing a higher number of Treg cells.

Recent meta-analyses of human and animal studies support the benefit of probiotics in the treatment of MS and EAE. Despite these benefits, questions remain about the effectiveness and potential side effects of probiotics. For example, several studies have implicated probiotic administration with septicemia, and patients at highest risk are those who are immunosuppressed and more likely to try probiotics in hopes of improving gut health (Oggioni et al., 1998; Lherm et al., 2002; Cassone et al., 2003; Ledoux et al., 2006). As indicated in several studies, co-administration of DMTs and probiotics may not necessarily increase the efficacy of disease amelioration. Thus, larger prospective studies are needed to understand the interactions between probiotics and DMTs and define what are the optimal windows of time during disease pathogenesis to administer probiotics.

Another important caveat to current probiotic studies is that most of the human studies have been done in Relapsing Remitting MS (RRMS) and no studies are currently available to evaluate the role of probiotics in patients with clinically isolated syndrome (CIS), primary and secondary progressive disease (SPMS). Further, most of the human studies have been performed primarily in females, and more inclusive studies are needed to define probiotic use in both sexes. Additionally, EAE studies have demonstrated that not all species of a genera may be beneficial and co-administration of different species of a single or multiple genera can have important interactions that may produce unexpected effects. To answer these emerging questions in probiotic research, detailed mechanistic studies are needed in animal models of EAE to best understand how individual strains and combined strains of probiotics act together in age-, sex-, and disease-specific patterns.

### Fecal microbiome transplant

Given the importance of the gut microbiome in MS pathology and the known dysbiotic microbiota patterns in MS patients, FMT may potentially offer a promising future therapeutic approach for the treatment of MS.

## Fecal microbiome transplant studies in multiple sclerosis

Fecal microbiome transplants have been successfully implemented in treating *Clostridioides difficile* diarrhea when no other treatments could help patients (Quraishi et al., 2017). In MS patients, several small studies have demonstrated the potential of the FMT therapeutic option (Borody et al., 2011; Makkawi et al., 2018; Engen et al., 2020). In these studies, FMT administered to MS patients with constipation not only improved patients’ bowel movement regularity but was also correlated with patients’ ability to discontinue urinary catheter use and regain the ability to walk as well as experience improvements in EDSS. Surprisingly, these neurological gains were sustained long-term.

## Fecal microbiome transplant studies in experimental autoimmune encephalomyelitis

In animal studies, FMT has been demonstrated to alter the course of EAE in recipients with transfer of microbiota either from animal or patient donors (Cekanaviciute et al., 2017; Mangalam et al., 2017; Wang et al., 2021). For example, transfer of Prevotella from MS donors to animal recipients resulted in decreased incidence of EAE (Mangalam et al., 2017). In another study, FMT from healthy mice to mice induced with EAE led to improvement in EAE scores and correlated with an increase in the abundance of *Firmicutes* and *Proteobacteria* and a decrease in the abundance of *Bacteroides* and *Actinobacteria* (Wang et al., 2021). In addition, FMT from un-treated control animals to mice with EAE led to restoration of the altered intestinal microbiota diversity in EAE mice as well as decreased activation of microglia and astrocytes and improved BBB integrity (Li et al., 2020). In another study, FMT from animals on intermittent fasting (IF) diet to naiive mice transferred the protective effects of the IF diet and ameliorated the EAE course in the recipients (Cignarella et al., 2018). Intriguingly, Boehme et al. (2021) recently demonstrated that transfer of microbiome from young to old animals can drastically reverse age related immune and neurological processes as well as cognitive behaviors, suggesting an immensely exciting avenue for affecting neuroinflammation and potentially neurodegeneration.

Mechanistically, fecal microbiome transplants may be immunoprotective in MS and EAE *via* several non-mutually exclusive mechanisms, such as downstream actions of bacterial molecules, metabolites, or evenpeptides from the bacterial wall reaching the CNS and having anti-inflammatory effects (Quévrain et al., 2016; Erturk-Hasdemir et al., 2021; Elsayed et al., 2022; Gabanyi et al., 2022). For example, fecal microRNAs, which are commonly present in the gut lumen and feces of humans and animals have been demonstrated to ameliorate EAE *via* expansion of *Akkermansia muciniphila* and dendritic cell (DC)-mediated Treg differentiation (Liu et al., 2019). In turn, components of microbacterial cell walls, such as peptidoglycan, which are persistently shed from microbacteria, are found in the brain and can interact with Nod-like receptors to influence neural activity and neuroinflammation (Gabanyi et al., 2022). In addition, bacterial products of microbiota, such as *Faecalibacterium prausnitzii*, have been shown to modulate autoimmunity by producing microbial anti-inflammatory molecule (MAM), which downregulates activation of the nuclear factor (NF)-κB pathway in a dose-dependent manner (Quevrain et al., 2016).

Overall, the collective data from animal and human studies suggest that FMT could be a viable therapeutic for MS. Although this approach is very exciting, multiple questions will need to be answered regarding efficacy and potential side effects prior to FMT becoming a mainstream treatment for MS. For example, important questions include (1) at what stage of MS disease process should FMT be offered to patients–i.e., CIS vs. RRMS vs. progressive MS; (2) should FMTs be age and sex- matched between donors and recipients; (3) what are the long term positive and negative effects of FMT; (4) do FMTs have to be performed recurrently or would one time suffice; (5) how should diet be adjusted post-FMT; and (6) what type of monitoring should be done to control for efficacy and side effects of FMT. Further scientific work will need to be initially performed in animal models followed by human studies to better understand the effects of FMT on MS pathogenesis prior to undertaking larger trials in patients.

### Dietary factors

#### Dietary fatty acids

Dietary fat has been studied in relation to MS for decades. In 1950, Swank observed higher rates of MS in inland populations of Norway with higher saturated fat intakes compared to lower rates in coastal communities consuming diets rich in polyunsaturated fatty acids (PUFAs) from seafood (Swank, 1950). Since then, multiple epidemiological and experimental studies have explored the potential protective and detrimental effects of FAs in MS.

Fatty acids are classified into short [1–5 carbon (C) atoms], medium (6–12C), and long fatty acids 14–22C, termed SCFAs, MCFAs, and LCFAs, respectively. The double bond number categorizes FAs as saturated vs. mono- or poly-unsaturated (M/PUFAs). Whereas many FAs are acquired from diet, SCFAs are primarily made *via* bacterial carbohydrate fermentation. SCFAs, such as acetate (C2), propionate (C3), and butyrate (C4) play an important role in the communication between the gut microbiome, immune, and nervous systems and in modulating gut-blood barrier integrity (Cummings et al., 1987; Haase et al., 2018; Saresella et al., 2020). MCFAs, in particular lauric acid, may be more pro-inflammatory, based on studies demonstrating influence on differentiation, proliferation, and CNS migration of Th1 and Th17 cells (Haghikia et al., 2015). LCFAs include PUFAs, such as omega-6 FA’s [e.g., linoleic acid (LNA, 18C)] and arachidonic acid (ARA, C20), and omega-3 FA’s [e.g., eicosatetraenoic acid (EPA 20C), and docosahexaenoic acid (DHA, 22C). LCFAs are known to directly modulate the phospholipid bilayer (Schumann et al., 2011). Although various fatty acids can impact the immune system, most MS research has focused on SCFAs and PUFAs.

## Short-chain fatty acid studies in multiple sclerosis

Several studies in MS patients have revealed decreased SCFAs in fecal and/or serum samples of MS patients with associations between SCFAs and peripheral or CNS-related inflammation. For example, Saresella et al. (2020) reported that low butyric acid concentration in a cohort of 38 RRMS and SPMS patients was correlated with higher lipopolysaccharide and alterations of gut barrier permeability. In another study of 30 patients with CIS or RRMS, the decrease in propionate levels in the serum was correlated with lower frequencies of T follicular regulatory cells (Trend et al., 2021). Similarly, Zeng et al. (2019) reported a decrease in acetate, propionate, and butyrate in a Chinese cohort of 34 MS patients compared to healthy controls linked to T follicular regulatory cell frequencies. Indeed, lower SCFA levels have been reported in multiple studies in different countries (Park et al., 2019; Zeng et al., 2019; Takewaki et al., 2020). Clinical trials in MS patients have demonstrated that supplementation with SCFAs, such as propionic acid, in combination with disease modifying therapies led to reduction in relapse rates and brain atrophy in MS patients compared to patients who did not receive propionic acid (Duscha et al., 2020).

Interestingly, some studies have documented higher levels of SCFA’s, such as acetate in MS patients. For example, a study of 63 pregnant MS patients demonstrated an increase in serum acetate levels in MS patients compared to non-MS patients (Becker et al., 2021). Higher ratios of propionate to acetate correlated with non-relapsing disease activity (Becker et al., 2021). Similarly, Pérez-Pérez et al. (2020) also found higher levels of acetate in the plasma of 46 RRMS patients with high disability scores (≥5.0 on the EDSS) compared to healthy controls (Zeng et al., 2019).

Yet, other human studies have not found a correlation between SCFA’s and MS. For instance, Becker et al. (2021), examining 41 RRMS patients (70.7% female, 29.3% male) and 35 healthy controls (37.1% female, 62.9% male) did not find significant differences in acetate, propionate, and butyrate levels between MS patients and controls (Trend et al., 2021).

Although some of these results may appear conflicting, there are important differences in study designs and patient populations in MS patient studies. Some studies measured SCFAs in fecal matter while others collected blood plasma or serum. In other autoimmune disorders, such as irritable bowel syndrome (IBD), there have been discrepancies reported between SCFA levels in blood vs. feces (Park et al., 2019). Becker et al. (2021) noted a difference in fecal SCFA levels between men and women, with men having overall higher levels of acetate, propionate, and butyrate compared to women. The sex makeup of patient populations could therefore also be a factor impacting the statistical significance of SCFA shifts. Additionally, geographical location, MS subtype, disease severity, immunomodulatory therapy status, and length of time since diagnosis differ across studies, which are all factors that could contribute to variability of SCFA presence and effects.

## Short-chain fatty acid studies in experimental autoimmune encephalomyelitis

Experimental autoimmune encephalomyelitis studies have provided evidence that SCFA modulation affects EAE severity and immune cell phenotypes. For example, B6 mice on a high fiber diet had significantly higher acetate and propionate production in the cecum, and reduced immune cell infiltration into the spinal cord (Mizuno et al., 2017). Arpaia et al. (2013) also observed that B6 mice fed a butyrylated starch diet had significantly higher colonic Treg generation than mice fed a control starch diet, suggesting that increased abundance of SCFAs, specifically butyrate, may dampen EAE severity by shifting T cell phenotypes. Additional studies have supported the claim that butyrate ingestion reduces EAE symptoms and spinal cord pathology and can promote Treg differentiation even under non-EAE conditions (Furusawa et al., 2015).

Other studies have focused more specifically on the effects of propionate administration. Duscha et al. (2014) found that propionic acid can reduce EAE severity and spinal cord damage in B6 mice when administered prior to EAE induction. However, in this study, propionic acid was ineffective when administered after mice had already started displaying EAE symptoms. Similarly, Haghikia et al. (2015) documented similar results by administering propionate either on the day of induction or at the time of EAE symptom onset. Only propionate ingestion at the time of EAE induction resulted in significant symptom reduction compared to EAE-induced mice on a normal chow diet. This subset of mice also had reduced white matter demyelination in the spinal cord and higher axonal density compared to controls. Additionally, propionate significantly increased relative axonal density compared to controls even in the group that did not receive propionate until the time of disease onset. Propionate supplementation, like butyrate, can result in an increased number of Tregs in the lymph nodes, which could be pivotal contributors to preserving axonal integrity during the MS disease course (Mizuno et al., 2017).

## Polyunsaturated fatty acid studies in multiple sclerosis

Potential ameliorative aspects of PUFAs have been demonstrated in several patient studies, starting with reports by Swank (1950), identifying lower rates of MS in association with omega-3 PUFA consumption from fish in Nordic coastal regions compared to farming areas (Swank et al., 1952). In a further long-standing study of 35 year follow-up of MS patients, Swank and Goodwin (2003) demonstrated that MS patients on a low-saturated fat diet supplemented with cod liver and vegetable oils (rich in PUFAs), led to benefits in mortality, relapse severity, and disability progression. In the 1970’s, in a prospective double-blind controlled dietary study in England, 45 female and 30 male MS patients were followed for 2 years (Millar et al., 1973). In this study, Millar et al. (1973) found that supplementation with linoleic acid lead to less frequent and less severe relapse rates in MS patients, although it did not affect long-term clinical progression. More recently, positive results with PUFA intake and supplementation were replicated in an international study of 1493 MS patients (82.3% female, 17.7% male). In this study, Jelinek et al. (2013) demonstrated that flaxseed oil supplementation and fish consumption three times per week were correlated with reduced relapse rates and disability progression, as well as with higher quality of life. Similarly, another international epidemiological survey study from 57 countries of 2,469 people with MS (82.2% female and 17.8% male) found a 44% lower odds of relapse in patients supplemented with plant-based omega 3 supplements (Jelinek et al., 2016). In one of the largest cohorts, the Nurses’ Health Studies (I: 80,920 women and II 94,511 women), a total of 479 women with MS were followed over 4 years with food diary questionnaires. This study reported that higher PUFA intake at baseline was linked with a lower risk of MS and an inverse association of MS risk with linolenic acid (Bjørnevik et al., 2017). Benefits of omega-3 PUFAs have also been demonstrated in a year-long intervention study in 31 RRMS patients, with findings that patients on a low fat diet combined with omega 3 PUFA supplementation had significantly reduced relapse rates in correlation with improved fatigue, and other scores on the Physical Component Scale and the Mental Health Inventory (Weinstock-Guttman et al., 2005).

Other patient studies did not replicate positive PUFA results. In a double-blind controlled trial of 312 MS patients (101 males and 211 females) treated with linoleic acid there was no significant benefit of PUFA supplementation on number of MS relapses (Bates et al., 1989). In this study, lower doses of linoleic acid were administered compared to a similar study by Millar et al. (1973). In another study of 20 MS patients supplemented daily with fish oil containing EPA and DHA, despite a reduction in tumor necrosis factor (TNF)-α, IL-1b, IL-6, and NO over 1 year of treatment, no effects were noted on EDSS or relapse rate (Ramirez-Ramirez et al., 2013). Likewise, Torkildsen et al. (2012) did not find a difference in gadolinium enhancing lesions or relapse rate in a cohort of 92 RRMS patients supplemented with EPA and DHA omega-3 PUFAs over a course of 24 months. While this study was one of the few to evaluate brain imaging, confounding factors included patients not being treated on any DMTs for up to 4 months of the study, and the supplements included oleic acid, which is known to have more inflammatory effects (Torkildsen et al., 2012).

## Polyunsaturated fatty acid studies in experimental autoimmune encephalomyelitis

Experimental autoimmune encephalomyelitis models have largely supported the benefits of PUFAs in neuroinflammation and have aided in elucidating the mechanism of action behind amelioration due to PUFA supplementation. For example, administration of 50 or 250 mg/kg body weight daily of the triacylglycerol form of DHA beginning 15 days prior to EAE induction and continuing for 41 days in female B6 mice (*n* = 10), resulted in reduced disease-associated weight loss compared to controls, and the 250 mg/kg group exhibited significant reductions in EAE scores (Mancera et al., 2017). Further, DHA treated animals demonstrated a decrease in microglial susceptibility to oxidative stress toxicity along with a reduction in inflammatory cytokines, TNF-α and IL-6 (Mancera et al., 2017). In a similar study, B6 female mice (*n* = 15) were fed a diets containing 0.3 or 1% phospholipid DHA (PL-DHA) or 0.3 or 1% triacylglycerol DHA (TAG-DHA) beginning 4 weeks prior to EAE induction with MOG (Adkins et al., 2019). The 1% PL-DHA-fed group exhibited significantly higher DHA concentration in the brain compared to controls, indicating that this form of DHA may be more bioavailable. Analysis of the EAE scores for each treatment group individually compared to controls yielded no significant lowering of EAE scores, but combined analysis of the DHA-treated groups revealed significant lowering of scores compared to controls. Additionally, the 1% TAG-DHA significantly reduced ARA in the brains of mice, which is associated with MS pathology and CNS inflammation (Adkins et al., 2019). In a smaller study, two groups female B6 mice (*n* = 4/group) were randomized to either a 5% EPA diet or a control diet beginning 2 weeks prior to MOG EAE induction and continuing 1 week after. The group on this EPA diet demonstrated a significant reduction in both EAE scores and infiltration of the spinal cord by lymphocytes. Following EAE, the researchers analyzed tissues to test the theory that EPA ameliorates EAE *via* the upregulation of peroxisome proliferator-activated receptor (PPAR) activity. They found significantly higher expression of PPARs in CD4 + infiltrates from murine CNS of EAE mice compared to controls, whereas peripheral CD4 + T cells did not exhibit a significant difference, except for PPARγ, which shows the highest affinity for EPA (Unoda et al., 2013).

The cuprizone model of demyelination has been used to explore the beneficial effects of PUFAs on myelination independent of their immunomodulatory effects. In cuprizone-fed mice, supplementation with omega-3 PUFAs in the form of salmon was shown to be significantly effective at preventing demyelination and reducing lesion volume compared to cod liver and soybean oil, despite the cod liver oil containing higher concentrations of EPA and DHA. Additionally, the salmon and soybean oil groups exhibited significantly more remyelination upon cuprizone discontinuation than the cod liver oil group, but no significant difference between the two was observed (Torkildsen et al., 2009). Other EAE studies have demonstrated that EPA can affect T cell differentiation and potential for remyelination. For example, rats treated with EPA demonstrated increased PLP in the medulla and cerebellum, as well as increased MOG and MBP mRNA transcripts (Salvati et al., 2008), likely due to PLP promoter containing *cis*-element(s) which are positively regulated by EPA (Salvati et al., 2004).

### High salt diet

With increased popularity of Westernized “fast food” diets around the world, there is a higher exposure to sodium chloride (salt), sometimes as high as 100 times higher than in homemade meals (Appel et al., 2011). Higher dietary salt consumption has been associated with both systemic diseases, such as hypertension (Wilck et al., 2017), renal disease (van den Berg et al., 2012), and cancer (Wu et al., 2021) as well as with autoimmune diseases such as lupus (Scrivo et al., 2017) and rheumatoid arthritis (Sundström et al., 2015). Nevertheless, there is also potential for benefit from high salt diet. For example, high salt preparations can benefit patients with low intravascular volume, hypotension, dysautonomia, high intracranial pressure, cystic fibrosis, and sinusitis (Johnson et al., 2010; Ropper, 2012; Hoegger et al., 2014; Chong et al., 2016; Pfortmueller and Schefold, 2017). Also, intravenous (IV) injection of salt solutions is frequently used in the hospital setting to rehydrate patients and treat hyponatremia (Moritz and Ayus, 2010).

## High salt diet studies in multiple sclerosis

The question of whether high salt diet (HSD) is detrimental or beneficial in MS has been evaluated both in human and animal EAE studies. One adult MS study examined RRMS patients, demonstrating that intake of HSD was associated with approximately four times greater rate of relapse and higher T2 lesion load compared to low-salt intake (Farez et al., 2015). Of note, the researchers used spot urine measurements to gauge sodium consumption and only had cohorts of 70 and 52 patients, respectively. Other studies that used more extensive sampling and larger sample sizes found no correlation between sodium intake and MS risk (Cortese et al., 2017) or progression (Fitzgerald et al., 2017). As mentioned in an editorial by Koch-Henriksen and Lauer (2017), the study by Cortese et al. (2017) can only be generalized to a subset of women and not to men since only the data collected were limited to women nurses. However, the enormous sample size of this prospective study supports the idea that, at least in adulthood, HSD does not increase the risk of developing MS.

Similarly to the larger adult studies, in pediatric MS studies, there was not an association between HSD and MS onset (McDonald et al., 2016) or time to relapse (Nourbakhsh et al., 2016). Importantly, however, both studies only used the Block Kids Food Screener questionnaire and did not collect any biological measurements. Therefore, there may be value in collecting 24-h urine samples from the pediatric population or performing a wide-scale, prospective longitudinal study beginning during childhood to examine if a prolonged HSD earlier in development correlates with MS risk. Additionally, no human MS studies have examined the interplay between sodium intake and genetics or gut microbiota.

## High salt diet studies in experimental autoimmune encephalomyelitis

Compared to human MS studies, investigations in most animal models of MS have supported a detrimental effect of HSD in EAE disease progression with evidence of increased Th17 cell (Kleinewietfeld et al., 2013; Wu et al., 2013) and macrophage infiltration (Hucke et al., 2016) into the CNS, enhanced production of pro-inflammatory cytokines, such as IL-17, TNF-alpha, IL-10, and GM-CSF (Hucke et al., 2016), and an increase in BBB permeability (Krementsov et al., 2015). Interestingly, EAE studies with HSD have also highlighted the important role of gut microbiota in MS disease pathogenesis. Specifically, HSD led to depletion of *L. murinus* and butyrate with an increase in Th17 cells in mice (Wilck et al., 2017; Miranda et al., 2018). In turn, supplementation with *L. murinus* or *L. reuteri* ameliorated HSD-induced EAE exacerbation (Wilck et al., 2017).

However, not all EAE studies have been congruent on the effects of HSD. In a recent study of HSD effects in a spontaneous EAE model, it was found that although HSD led to an increase in Th17 cells, there was an overall suppressed development of EAE in HSD-treated animals compared to controls (Na et al., 2021). These studies demonstrate that the differing results in EAE HSD investigations depend on the type of model evaluated (i.e., induced EAE vs. spontaneous EAE), mode of HSD delivery (i.e., water vs. pellets), and the age and sex of animals.

Despite limitations of human and animal studies, the collective studies implicate HSD in MS disease pathogenesis with important mechanistic insights on the immune effects of HSD. Future studies on HSD will need to compare patient cohorts by sex, age, and genetics and incorporate these factors in animal models of MS. Importantly, since HSD can lead to hypertension, an independent risk factor for MS exacerbation, future studies will also need to control for hypertension.

### Alcohol

Alcohol is less commonly thought of as a dietary factor, although it is widely consumed in many cultures and by the MS patient population. In fact, some studies have estimated that as many as 40% of patients diagnosed with MS drink excessively (Beier et al., 2014).

## Alcohol studies in multiple sclerosis

Interestingly, alcohol has been found to ameliorate inflammation across multiple autoimmune diseases (Caslin et al., 2021). Studies in MS patients have also demonstrated that alcohol can modulate MS risk. Four large epidemiological studies support an inverse relationship between alcohol consumption and risk of MS. In a Swedish cohort of 745 MS patients and 1,761 controls consuming low to moderate dose alcohol, there was a statistically significant dose-dependent inverse association between alcohol intake and risk of MS in both men and women (Hedström et al., 2014). Another cohort study of 1,717 MS patients and 4,685 controls in a Danish MS Biobank study similarly found an inverse relationship between alcohol consumption and MS incidence (Andersen et al., 2019). In addition, in a cohort of 923 MS patients at the Brigham and Women’s Hospital, light-moderate drinking was associated with a decrease in MS disease activity. Lastly, in a cohort of 272 MS patients and 151 healthy controls of patients at the University of Buffalo registry, alcohol consumption led to an increased age of disease onset. Further, patients older than 21 years old who consumed alcohol for up to 15 years had lower EDSS and higher gray matter volumes (GMV). GMV was greater in MS patients who had consumed alcohol for a period of 15 years or less after MS onset compared to non-drinkers or drinkers of greater than 15 years (Foster et al., 2012). In contrast, Pakpoor et al. (2014) evaluated 429 MS patients and 547,288 controls with alcohol dependence or abuse in a British database and reported that both alcohol abuse and dependence positively correlate with MS diagnosis, particularly in men. Lastly, in the Nurses’ Health Study of 258 primarily female MS patients and 238,371 controls, there was no correlation between alcohol consumption and MS risk (Massa et al., 2013). These studies suggest that dose of alcohol, country of residence and patient sex are likely important variables in alcohol’s effects on MS autoimmunity.

## Alcohol studies in experimental autoimmune encephalomyelitis

While several human epidemiological studies have demonstrated the potential benefit of alcohol in MS onset and progression, alcohol’s mechanism of action in MS neuroinflammation remains to be elucidated. Several EAE studies have now reported benefits of moderate alcohol diet in EAE neuroinflammation. For example, Azizov et al. (2020) found that chronic moderate alcohol consumption resulted in symptom reduction and lower disease incidence in a mouse model of EAE. Similarly, Caslin et al. (2019) demonstrated ameliorating effects of moderate alcohol in EAE. Interestingly, these results were sex-specific, such that the moderate alcohol-consuming males experienced a more significant disease amelioration compared to alcohol-consuming females and control groups (Caslin et al., 2019). Mechanistically, this study reported higher levels of microbiota with immune regulatory roles, including *Turicibacter, Akkermansia, Prevotella*, and *Clostridium* as well as lower number of microglia in alcohol consuming males compared to control-diet consuming males and female groups (Caslin et al., 2019).

Given that many MS patients consume alcohol and there are currently no accepted guidelines for alcohol use in MS, further studies are necessary to define alcohol’s impact on the immune system, gut microbiome, and the nervous system, to better understand alcohol’s dose-related effects in MS. While EAE modeling may not 100% recapitulate human physiology, alcohol is known to modulate the immune system similarly in humans and mice and prior studies have defined alcohol dosing translation across species (Nair and Jacob, 2016; Pruett et al., 2020). Further, examining this question in EAE animal models is critical since it would be unethical to prescribe chronic alcohol to vulnerable patient populations. Ultimately, a better understanding of alcohol’s neuroprotective effects could define new alcohol-independent therapeutic targets in MS.

## Conclusion and future directions

Accumulating evidence from gut microbiome studies of MS patients and EAE animal models has raised the optimism for the possibility of treating MS with gut bacterial modulatory approaches, such as supplementation of probiotic, antibiotics and SCFAs, FMT, and direct manipulation of diet. The gut microbiome approach to therapeutics may be of particular importance since current DMTs primarily target the adaptive immune system, are costly, have side effects, need to be continued indefinitely, and do not target remyelination. Thus, there is an unmet clinical need for developing more cost-efficient therapies that target the gut microbiome with potential for remyelinating the CNS and with sustained long-term efficacy and fewer side effects. Nevertheless, further detailed mechanistic studies will need to be performed in both EAE animal models and MS patients before microbiome-specific therapies are widely deployed given the observed variability between studies.

As exemplified by the recent iMSMS Consortium study (Zhou et al., 2022), there are significant differences in the gut microbiome species and gut-derived metabolites in patients across MS disease states, countries of residence, and DMTs. Thus, in future humans studies, it will be important to ensure that studies are sufficiently powered, include both males and females, compare patients across disease states (RRMS vs. SPMS vs. CIS), and take into account patient age, latitude of residence, and treatment with DMTs. Based on the iMSMS Consortium study results (Zhou et al., 2022), demonstrating that diet alone does not account for microbiota diversity, future MS microbiome studies will also need to account not only for patient dietary habits but also for other environmental exposures, such as water or air, that may influence the gut microbiome. In addition, consistency between sampled tissues will be critical, as there are known differences in gut bacterial and FA composition between serum and fecal samples. Lastly, it will be important to sample multiple gastrointestinal compartments, such as the oral, esophageal, intestinal, and colonic compartments, as studies have suggested differences in the microbial composition of these regions (Martinez-Guryn et al., 2019). While no one EAE model can approximate the complex nature of MS pathophysiology, the development of a myriad of EAE models over the last decade has tremendously expanded opportunities for examining different aspects of MS pathophysiology and specifically microbiome-targeted therapeutics. To continue improving precision and translatability of EAE models, several strategies could be implemented in future EAE research. For example, inclusion of wild caught animals and humanized mice could help to better recapitulate the immune system and gut microbiome of humans. Given the sex bias in MS, inclusion of SRY transgenic animals (Arnold and Burgoyne, 2004) in more studies would increase knowledge on how MS pathophysiology affects males and females differently. In addition, including non-human primates, such as marmosets, in EAE research could offer a more precise preclinical platform for evaluation of DMTs, given the closer immunological milieu of marmosets and humans (A’t Hart et al., 2005). Non-human primate use would also allow for more facile opportunities to evaluate blood and cerebrospinal fluid as well as perform electrophysiological studies.

Interestingly, development of recent bioinformatic models, such as Computer Aided Drug Design and structure-activity relationship models (Doke and Dhawale, 2015), has created a new way to model biological activity of potential new therapeutics at early stages ahead of animal and human studies. However, it will continue to be critical to include human cell line studies for early-stage screening and ultimately design well-powered, longitudinal, age, and sex-matched randomized controlled MS patient trials to test emerging therapeutics and their potential adverse events most directly.

## Author contributions

EM: study concept and design, drafting and revision of the manuscript for content, generation of figure, preparation of manuscript for journal submission, and funding for the study. JP: drafting and revision of the manuscript, generation of the tables, and participated in study design. CF: drafting and revision of the manuscript and participated in study design. All authors contributed to the article and approved the submitted version.

## References

[B1] Adkins Y. Soulika A. M. Mackey B. Kelley D. S. (2019). Docosahexaenoic acid (22: 6n-3) ameliorated the onset and severity of experimental autoimmune encephalomyelitis in mice. *Lipids* 54 13–23. 10.1002/lipd.12130 30762234

[B2] Ahlgren C. Odén A. Lycke J. (2011). High nationwide prevalence of multiple sclerosis in Sweden. *Mult. Scler. J.* 17 901–908. 10.1177/1352458511403794 21459810

[B3] Alfredsson L. Olsson T. (2019). Lifestyle and environmental factors in multiple sclerosis. *Cold Spring Harb. Perspect. Med.* 9:a028944. 10.1101/cshperspect.a028944 29735578 PMC6444694

[B4] Almasi-Hashiani A. Sahraian M. A. Eskandarieh S. (2020). Evidence of an increased prevalence of multiple sclerosis: A population-based study of Tehran registry during 1999-2018. *BMC Neurol.* 20:169. 10.1186/s12883-020-01747-8 32359352 PMC7195783

[B5] Alonso A. Jick S. S. Jick H. Hernán M. A. (2006). Antibiotic use and risk of multiple sclerosis. *Am. J. Epidemiol.* 163 997–1002. 10.1093/aje/kwj123 16597708

[B6] Amor S. Groome N. Linington C. Morris M. M. Dornmair K. Gardinier M. V. (1994). Identification of epitopes of myelin oligodendrocyte glycoprotein for the induction of experimental allergic encephalomyelitis in SJL and Biozzi AB/H mice. *J. Immunol.* 153 4349–4356. 7525700

[B7] Andersen C. Søndergaard H. B. Bang Oturai D. Laursen J. H. Gustavsen S. Larsen N. K. (2019). Alcohol consumption in adolescence is associated with a lower risk of multiple sclerosis in a Danish cohort. *Mult. Scler. J.* 25 1572–1579. 10.1177/1352458518795418 30124094

[B8] Appel L. J. Frohlich E. D. Hall J. E. Pearson T. A. Sacco R. L. Seals D. R. (2011). The importance of population-wide sodium reduction as a means to prevent cardiovascular disease and stroke: A call to action from the American Heart Association. *Circulation* 123 1138–1143. 10.1161/CIR.0b013e31820d0793 21233236

[B9] Arnold A. P. Burgoyne P. S. (2004). Are XX and XY brain cells intrinsically different? *Trends Endocrinol. Metab.* 15 6–11. 10.1016/j.tem.2003.11.001 14693420

[B10] Arpaia N. Campbell C. Fan X. Dikiy S. van der Veeken J. deRoos P. (2013). Metabolites produced by commensal bacteria promote peripheral regulatory T-cell generation. *Nature* 504 451–455. 10.1038/nature12726 24226773 PMC3869884

[B11] Ascherio A. Munger K. (2008). Epidemiology of multiple sclerosis: From risk factors to prevention. *Semin. Neurol.* 28 17–28. 10.1055/s-2007-1019126 18256984

[B12] A’t Hart B. Bauer J. Brok H. P. Amor S. (2005). Non-human primate models of experimental autoimmune encephalomyelitis: Variations on a theme. *J. Neuroimmunol.* 168 1–12. 10.1016/j.jneuroim.2005.05.017 16023737

[B13] Azizov V. Dietel K. Steffen F. Dürholz K. Meidenbauer J. Lucas S. (2020). Ethanol consumption inhibits TFH cell responses and the development of autoimmune arthritis. *Nat. Commun.* 11:1998. 10.1038/s41467-020-15855-z 32332730 PMC7181688

[B14] Bargagli A. M. Colais P. Agabiti N. Mayer F. Buttari F. Centonze D. (2016). Prevalence of multiple sclerosis in the Lazio region, Italy: Use of an algorithm based on health information systems. *J. Neurol.* 263 751–759. 10.1007/s00415-016-8049-8 26886201 PMC4826660

[B15] Barnett M. Williams D. Day S. Macaskill P. McLeod J. (2003). Progressive increase in incidence and prevalence of multiple sclerosis in Newcastle, Australia: A 35-year study. *J. Neurol. Sci.* 213 1–6. 10.1016/s0022-510x(03)00122-9 12873746

[B16] Barrett E. Ross R. O’Toole P. W. Fitzgerald G. F. Stanton C. (2012). γ-Aminobutyric acid production by culturable bacteria from the human intestine. *J. Appl. Microbiol.* 113 411–417. 10.1111/j.1365-2672.2012.05344.x 22612585

[B17] Bates D. Cartlidge N. French J. Jackson M. J. Nightingale S. Shaw D. A. (1989). double-blind controlled trial of long chain n-3 polyunsaturated fatty acids in the treatment of multiple sclerosis. *J. Neurol. Neurosurg. Psychiatry* 52 18–22. 10.1136/jnnp.52.1.18 2540285 PMC1032650

[B18] Becker A. Abuazab M. Schwiertz A. Walter S. Faßbender K. C. Fousse M. (2021). Short-chain fatty acids and intestinal inflammation in multiple sclerosis: Modulation of female susceptibility by microbial products? *Autoimmun. Highlights* 12:7. 10.1186/s13317-021-00149-1 33827656 PMC8028206

[B19] Beier M. D’Orio V. Spat J. Shuman M. Foley F. W. (2014). Alcohol and substance use in multiple sclerosis. *J. Neurol. Sci.* 338 122–127. 10.1016/j.jns.2013.12.029 24411661

[B20] Berer K. Gerdes L. A. Cekanaviciute E. Jia X. Xiao L. Xia Z. (2017). Gut microbiota from multiple sclerosis patients enables spontaneous autoimmune encephalomyelitis in mice. *Proc. Natl. Acad. Sci. U.S.A.* 114 10719–10724. 10.1073/pnas.1711233114 28893994 PMC5635914

[B21] Berer K. Mues M. Koutrolos M. Rasbi Z. A. Boziki M. Johner C. (2011). Commensal microbiota and myelin autoantigen cooperate to trigger autoimmune demyelination. *Nature* 479 538–541. 10.1038/nature10554 22031325

[B22] Bettelli E. Baeten D. Jäger A. Sobel R. A. Kuchroo V. K. (2006). Myelin oligodendrocyte glycoprotein–specific T and B cells cooperate to induce a Devic-like disease in mice. *J. Clin. Investig.* 116 2393–2402. 10.1172/JCI28334 16955141 PMC1555670

[B23] Bettelli E. Pagany M. Weiner H. L. Linington C. Sobel R. A. Kuchroo V. K. (2003). Myelin oligodendrocyte glycoprotein-specific T cell receptor transgenic mice develop spontaneous autoimmune optic neuritis. *J. Exp. Med.* 197 1073–1081. 10.1084/jem.20021603 12732654 PMC2193967

[B24] Bhuiyan P. Chen Y. Karim M. Dong H. Qian Y. (2021). Bidirectional communication between mast cells and the gut-brain axis in neurodegenerative diseases: Avenues for therapeutic intervention. *Brain Res. Bull.* 172 61–78. 10.1016/j.brainresbull.2021.04.010 33892083

[B25] Bjørnevik K. Chitnis T. Ascherio A. Munger K. L. (2017). Polyunsaturated fatty acids and the risk of multiple sclerosis. *Mult. Scler. J.* 23 1830–1838. 10.1177/1352458517691150 28156186 PMC5494026

[B26] Boehme M. Guzzetta K. E. Bastiaanssen T. F. S. van de Wouw M. Moloney G. M. Gual-Grau A. (2021). Microbiota from young mice counteracts selective age-associated behavioral deficits. *Nat. Aging* 1 666–676. 10.1038/s43587-021-00093-9 37117767

[B27] Borody T. Leis S. Campbell J. Torres M. Nowak A. (2011). Fecal microbiota transplantation (FMT) in multiple sclerosis (MS): 942. *Official J. Am. Coll. Gastroenterol. ACG* 106:S352.

[B28] Boussamet L. Rajoka M. S. R. Berthelot L. (2022). Microbiota, IgA and Multiple Sclerosis. *Microorganisms* 10:617. 10.3390/microorganisms10030617 35336190 PMC8954136

[B29] Brabb T. Goldrath A. W. von Dassow P. Paez A. Liggitt H. D. Goverman J. (1997). Triggers of autoimmune disease in a murine TCR-transgenic model for multiple sclerosis. *J. Immunol.* 159:497. 9200491

[B30] Braniste V. Al-Asmakh M. Kowal C. Anuar F. Abbaspour A. Tóth M. (2014). The gut microbiota influences blood-brain barrier permeability in mice. *Sci. Transl. Med.* 6:263ra158. 10.1126/scitranslmed.3009759 PMC4396848 25411471

[B31] Calvo-Barreiro L. Eixarch H. Ponce-Alonso M. Castillo M. Lebrón-Galán R. Mestre L. (2020). Commercial probiotic induces tolerogenic and reduces pathogenic responses in experimental autoimmune encephalomyelitis. *Cells* 9:906. 10.3390/cells9040906 32272791 PMC7226819

[B32] Caslin B. Maguire C. Karmakar A. Mohler K. Wylie D. Melamed E. (2019). Alcohol shifts gut microbial networks and ameliorates a murine model of neuroinflammation in a sex-specific pattern. *Proc. Natl. Acad. Sci. U.S.A.* 116 25808–25815. 10.1073/pnas.1912359116 31792189 PMC6925977

[B33] Caslin B. Mohler K. Thiagarajan S. Melamed E. (2021). Alcohol as friend or foe in autoimmune diseases: A role for gut microbiome? *Gut Microbes* 13:1916278. 10.1080/19490976.2021.1916278 34224314 PMC8259720

[B34] Cassone M. Serra P. Mondello F. Girolamo A. Scafetti S. Pistella E. (2003). Outbreak of Saccharomyces cerevisiae subtype boulardii fungemia in patients neighboring those treated with a probiotic preparation of the organism. *J. Clin. Microbiol.* 41 5340–5343. 10.1128/JCM.41.11.5340-5343.2003 14605200 PMC262466

[B35] Cekanaviciute E. Yoo B. B. Runia T. F. Debelius J. W. Singh S. Nelson C. A. (2017). Gut bacteria from multiple sclerosis patients modulate human T cells and exacerbate symptoms in mouse models. *Proc. Natl. Acad. Sci. U.S.A.* 114 10713–10718. 10.1073/pnas.1711235114 28893978 PMC5635915

[B36] Chen J. Chia N. Kalari K. R. Yao J. Z. Novotna M. Paz Soldan M. M. (2016). Multiple sclerosis patients have a distinct gut microbiota compared to healthy controls. *Sci. Rep.* 6:28484. 10.1038/srep28484 27346372 PMC4921909

[B37] Chen T. Noto D. Hoshino Y. Mizuno M. Miyake S. (2019). Butyrate suppresses demyelination and enhances remyelination. *J. Neuroinflamm.* 16:165. 10.1186/s12974-019-1552-y 31399117 PMC6688239

[B38] Chong L. Y. Head K. Hopkins C. Philpott C. Glew S. Scadding G. (2016). Saline irrigation for chronic rhinosinusitis. *Cochrane Database Syst. Rev.* 4:CD011995. 10.1002/14651858.CD011995.pub2 27115216 PMC8078614

[B39] Cignarella F. Cantoni C. Ghezzi L. Salter A. Dorsett Y. Chen L. (2018). Intermittent fasting confers protection in CNS autoimmunity by altering the gut microbiota. *Cell Metab.* 27 1222–1235.e6. 10.1016/j.cmet.2018.05.006 29874567 PMC6460288

[B40] Consonni A. Cordiglieri C. Rinaldi E. Marolda R. Ravanelli I. Guidesi E. (2018). Administration of bifidobacterium and lactobacillus strains modulates experimental myasthenia gravis and experimental encephalomyelitis in Lewis rats. *Oncotarget* 9 22269–22287. 10.18632/oncotarget.25170 29854277 PMC5976463

[B41] Cortese M. Yuan C. Chitnis T. Ascherio A. Munger K. L. (2017). No association between dietary sodium intake and the risk of multiple sclerosis. *Neurology* 89 1322–1329. 10.1212/WNL.0000000000004417 28842447 PMC5649760

[B42] Cosorich I. Dalla-Costa G. Sorini C. Ferrarese R. Messina M. J. Dolpady J. (2017). High frequency of intestinal TH17 cells correlates with microbiota alterations and disease activity in multiple sclerosis. *Sci. Adv.* 3:e1700492. 10.1126/sciadv.1700492 28706993 PMC5507635

[B43] Cryan J. F. Dinan T. G. (2012). Mind-altering microorganisms: The impact of the gut microbiota on brain and behaviour. *Nat. Rev. Neurosci.* 13 701–712. 10.1038/nrn3346 22968153

[B44] Cryan J. F. O’Mahony S. M. (2011). The microbiome-gut-brain axis: From bowel to behavior. *Neurogastroenterol. Motil.* 23 187–192. 10.1111/j.1365-2982.2010.01664.x 21303428

[B45] Cryan J. F. O’Riordan K. J. Sandhu K. Peterson V. Dinan T. G. (2020). The gut microbiome in neurological disorders. *Lancet Neurol.* 19 179–194. 10.1016/S1474-4422(19)30356-4 31753762

[B46] Cummings J. H. Pomare E. W. Branch W. J. Naylor C. P. Macfarlane G. T. (1987). Short chain fatty acids in human large intestine, portal, hepatic and venous blood. *Gut* 28 1221–1227. 3678950 10.1136/gut.28.10.1221 PMC1433442

[B47] Dantzer R. O’connor J. C. Freund G. G. Johnson R. W. Kelley K. W. (2008). From inflammation to sickness and depression: When the immune system subjugates the brain. *Nat. Rev. Neurosci.* 9 46–56. 10.1038/nrn2297 18073775 PMC2919277

[B48] De Paula M. L. Rodrigues D. H. Teixeira H. C. Barsante M. M. Souza M. A. Ferreira A. P. (2008). Genistein down-modulates pro-inflammatory cytokines and reverses clinical signs of experimental autoimmune encephalomyelitis. *Int. Immunopharmacol.* 8 1291–1297. 10.1016/j.intimp.2008.05.002 18602076

[B49] de Rosbo N. K. Mendel I. Ben-Nun A. (1995). Chronic relapsing experimental autoimmune encephalomyelitis with a delayed onset and an atypical clinical course, induced in PL/J mice by myelin oligodendrocyte glycoprotein (MOG)-derived peptide: Preliminary analysis of MOG T cell epitopes. *Eur. J. Immunol.* 25 985–993. 10.1002/eji.1830250419 7737302

[B50] Degenhardt A. Ramagopalan S. V. Scalfari A. Ebers G. C. (2009). Clinical prognostic factors in multiple sclerosis: A natural history review. *Nat. Rev. Neurol.* 5 672–682. 19953117 10.1038/nrneurol.2009.178

[B51] Doke S. K. Dhawale S. C. (2015). Alternatives to animal testing: A review. *Saudi Pharm. J.* 23 223–229. 10.1016/j.jsps.2013.11.002 26106269 PMC4475840

[B52] Duc D. Vigne S. Bernier-Latmani J. Yersin Y. Ruiz F. Gaïa N. (2019). Disrupting myelin-specific Th17 cell gut homing confers protection in an adoptive transfer experimental autoimmune encephalomyelitis. *Cell Rep.* 29 378–390.e4. 10.1016/j.celrep.2019.09.002 31597098

[B53] Duerkop B. A. Vaishnava S. Hooper L. V. (2009). Immune responses to the microbiota at the intestinal mucosal surface. *Immunity* 31 368–376. 10.1016/j.immuni.2009.08.009 19766080

[B54] Dunn S. E. Lee H. Pavri F. R. Zhang M. A. (2015). Sex-based differences in multiple sclerosis (Part I): Biology of disease incidence. *Curr. Top. Behav. Neurosci.* 26 29–56. 10.1007/7854_2015_371 25690593

[B55] Duscha A. Berg J. Thöne J. Demir S. Gold R. Haghikia A. (2014). Beneficial effects of short chain fatty acids on the course of experimental autoimmune encephalomyelitis. *J. Neuroimmunol.* 275:59. 10.1016/j.jneuroim.2014.08.154

[B56] Duscha A. Gisevius B. Hirschberg S. Yissachar N. Stangl G. I. Eilers E. (2020). Propionic acid shapes the multiple sclerosis disease course by an immunomodulatory mechanism. *Cell* 180 1067–1080.e16. 10.1016/j.cell.2020.02.035 32160527

[B57] Elsayed N. S. Aston P. Bayanagari V. R. Shukla S. K. (2022). The gut microbiome molecular mimicry piece in the multiple sclerosis puzzle. *Front. Immunol.* 13:972160. 10.3389/fimmu.2022.972160 36045671 PMC9420973

[B58] Engen P. A. Zaferiou A. Rasmussen H. Naqib A. Green S. J. Fogg L. F. (2020). Single-arm, non-randomized, time series, single-subject study of fecal microbiota transplantation in multiple sclerosis. *Front. Neurol.* 11:978. 10.3389/fneur.2020.00978 33013647 PMC7506051

[B59] Erny D. Hrabě de Angelis A. L. Jaitin D. Jaitin D. Wieghofer P. Staszewski O. (2015). Host microbiota constantly control maturation and function of microglia in the CNS. *Nat. Neurosci.* 18 965–977. 10.1038/nn.4030 26030851 PMC5528863

[B60] Erturk-Hasdemir D. Ochoa-Repa raz J. Kasper D. L. Kasper L. H. (2021). Exploring the gut-brain axis for the control of CNS inflammatory demyelination: Immunomodulation by *Bacteroides* fragilis’ polysaccharide A. *Front. Immunol.* 12:662807. 10.3389/fimmu2021 34025663 PMC8131524

[B61] Farez M. F. Fiol M. P. Gaitán M. I. Quintana F. J. Correale J. (2015). Sodium intake is associated with increased disease activity in multiple sclerosis. *J. Neurol. Neurosurg. Psychiatry* 86 26–31. 25168393 10.1136/jnnp-2014-307928 PMC12930402

[B62] Fitzgerald K. C. Munger K. L. Hartung H. P. Freedman M. S. Montalbán X. Edan G. (2017). Sodium intake and multiple sclerosis activity and progression in BENEFIT. *Ann. Neurol.* 82 20–29. 10.1002/ana.24965 28556498 PMC5555227

[B63] Forsythe P. Bienenstock J. (2010). Immunomodulation by commensal and probiotic bacteria. *Immunol. Invest.* 39 429–448. 20450286 10.3109/08820131003667978

[B64] Foster M. Zivadinov R. Weinstock-Guttman B. Tamaño-Blanco M. Badgett D. Carl E. (2012). Associations of moderate alcohol consumption with clinical and MRI measures in multiple sclerosis. *J. Neuroimmunol.* 243 61–68. 10.1016/j.jneuroim.2011.12.007 22261546

[B65] Fujino M. Funeshima N. Kitazawa Y. Kimura H. Amemiya H. Suzuki S. (2003). Amelioration of experimental autoimmune encephalomyelitis in Lewis rats by FTY720 treatment. *J. Pharmacol. Exp. Ther.* 305 70–77. 10.1124/jpet.102.045658 12649354

[B66] Furusawa Y. Obata Y. Hase K. (2015). Commensal microbiota regulates T cell fate decision in the gut. *Semin. Immunopathol.* 37 17–25. 10.1007/s00281-014-0455-3 25315350

[B67] Gabanyi I. Lepousez G. Wheeler R. Vieites-Prado A. Nissant A. Wagner S. (2022). Bacterial sensing *via* neuronal Nod2 regulates appetite and body temperature. *Science* 376:eabj3986. 10.1126/science.abj3986 35420957

[B68] Gharehkhani Digehsara S. Name N. Esfandiari B. Karim E. Taheri S. Tajabadi-Ebrahimi M. (2021). Effects of Lactobacillus casei Strain T2 (IBRC-M10783) on the Modulation of Th17/Treg and Evaluation of miR-155, miR-25, and IDO-1 Expression in a Cuprizone-Induced C57BL/6 Mouse Model of Demyelination. *Inflammation* 44 334–343. 10.1007/s10753-020-01339-1 32914363

[B69] Gödel C. Kunkel B. Kashani A. Lassmann H. Arumugam M. Krishnamoorthy G. (2020). Perturbation of gut microbiota decreases susceptibility but does not modulate ongoing autoimmune neurological disease. *J. Neuroinflamm.* 17:79. 10.1186/s12974-020-01766-9 32143718 PMC7060541

[B70] Gouider R. Mrabet S. Souissi A. Sghaier I. Kacem I. (2022). “Multiple Sclerosis in Migrants,” in *Neurology in Migrants and Refugees*, eds El Alaoui-Faris M. Federico A. Grisold W. (Berlin: Springer International Publishing), 189–200.

[B71] Goverman J. Woods A. Larson L. Weiner L. P. Hood L. Zaller D. M. (1993). Transgenic mice that express a myelin basic protein-specific T cell receptor develop spontaneous autoimmunity. *Cell* 72 551–560. 7679952 10.1016/0092-8674(93)90074-z

[B72] Haase S. Haghikia A. Wilck N. Müller D. N. Linker R. A. (2018). Impacts of microbiome metabolites on immune regulation and autoimmunity. *Immunology* 154 230–238. 10.1111/imm.12933 29637999 PMC5980218

[B73] Haghikia A. Jörg S. Duscha A. Berg J. Manzel A. Waschbisch A. (2015). Dietary fatty acids directly impact central nervous system autoimmunity *via* the small intestine. *Immunity* 43 817–829. 10.1016/j.immuni.2015.09.007 26488817

[B74] Harbo H. F. Gold R. Tintoré M. (2013). Sex and gender issues in multiple sclerosis. *Ther. Adv. Neurol. Disord.* 6 237–248. 10.1177/1756285613488434 23858327 PMC3707353

[B75] Harrington C. J. Paez A. Hunkapiller T. Mannikko V. Brabb T. Ahearn M. (1998). Differential tolerance is induced in T cells recognizing distinct epitopes of myelin basic protein. *Immunity* 8 571–580. 10.1016/s1074-7613(00)80562-2 9620678

[B76] He B. Hoang T. K. Tian X. Taylor C. M. Blanchard E. Luo M. (2019). Lactobacillus reuteri reduces the severity of experimental autoimmune encephalomyelitis in mice by modulating gut microbiota. *Front. Immunol.* 10:385. 10.3389/fimmu.2019.00385 30899262 PMC6416370

[B77] Hedström A. K. Hillert J. Olsson T. Alfredsson L. (2014). Alcohol as a modifiable lifestyle factor affecting multiple sclerosis risk. *JAMA Neurol.* 71 300–305. 24395432 10.1001/jamaneurol.2013.5858

[B78] Hiremath M. Saito Y. Knapp G. Ting J.-Y. Suzuki K. Matsushima G. (1998). Microglial/macrophage accumulation during cuprizone-induced demyelination in C57BL/6 mice. *J. Neuroimmunol.* 92 38–49. 10.1016/s0165-5728(98)00168-4 9916878

[B79] Hoegger M. J. Fischer A. J. McMenimen J. D. Ostedgaard L. S. Tucker A. J. Awadalla M. A. (2014). Impaired mucus detachment disrupts mucociliary transport in a piglet model of cystic fibrosis. *Science* 345 818–822. 10.1126/science.1255825 25124441 PMC4346163

[B80] Hoyles L. Snelling T. Umlai U.-K. Nicholson J. K. Carding S. R. Glen R. C. (2018). Microbiome–host systems interactions: Protective effects of propionate upon the blood–brain barrier. *Microbiome* 6:55. 10.1186/s40168-018-0439-y 29562936 PMC5863458

[B81] Hucke S. Eschborn M. Liebmann M. Herold M. Freise N. Engbers A. (2016). Sodium chloride promotes pro-inflammatory macrophage polarization thereby aggravating CNS autoimmunity. *J. Autoimmun.* 67 90–101. 10.1016/j.jaut.2015.11.001 26584738

[B82] Jangi S. Gandhi R. Cox L. M. Li N. von Glehn F. Yan R. (2016). Alterations of the human gut microbiome in multiple sclerosis. *Nat. Commun.* 7:12015. 10.1038/ncomms12015 27352007 PMC4931233

[B83] Jelinek G. A. De Livera A. M. Marck C. H. Brown C. R. Neate S. L. Taylor K. L. (2016). Associations of lifestyle, medication, and socio-demographic factors with disability in people with multiple sclerosis: An international cross-sectional study. *PLoS One* 11:e0161701. 10.1371/journal.pone.0161701 27560626 PMC4999178

[B84] Jelinek G. A. Hadgkiss E. J. Weiland T. J. Pereira N. G. Marck C. H. van der Meer D. M. (2013). Association of fish consumption and omega 3 supplementation with quality of life, disability and disease activity in an international cohort of people with multiple sclerosis. *Int. J. Neurosci.* 123 792–801. 10.3109/00207454.2013.803104 23713615 PMC3821380

[B85] Jiang J. Chu C. Wu C. Wang C. Zhang C. Li T. (2021). Efficacy of probiotics in multiple sclerosis: A systematic review of preclinical trials and meta-analysis of randomized controlled trials. *Food Funct.* 12 2354–2377. 10.1039/d0fo03203d 33629669

[B86] Johanson D. M. Goertz J. E. Marin I. A. Costello J. Overall C. C. Gaultier A. (2020). Experimental autoimmune encephalomyelitis is associated with changes of the microbiota composition in the gastrointestinal tract. *Sci. Rep.* 10:15183. 32938979 10.1038/s41598-020-72197-y PMC7494894

[B87] Johnson J. N. Mack K. J. Kuntz N. L. Brands C. K. Porter C. J. Fischer P. R. (2010). Postural orthostatic tachycardia syndrome: A clinical review. *Pediatr. Neurol.* 42 77–85. 10.1016/j.pediatrneurol.2009.07.002 20117742

[B88] Katz Sand I. Zhu Y. Ntranos A. Clemente J. C. Cekanaviciute E. Brandstadter R. (2019). Disease-modifying therapies alter gut microbial composition in MS. *Neurol. Neuroimmunol. Neuroinflamm.* 6:e517. 10.1212/NXI.0000000000000517 30568995 PMC6278850

[B89] Kaur H. Bose C. Mande S. S. (2019). Tryptophan metabolism by gut microbiome and gut-brain-axis: An *in silico* analysis. *Front. Neurosci.* 13:1365. 10.3389/fnins.2019.01365 31920519 PMC6930238

[B90] Keough M. B. Jensen S. K. Yong V. W. (2015). Experimental demyelination and remyelination of murine spinal cord by focal injection of lysolecithin. *Jove J. Vis. Exp.* 2015:e52679. 10.3791/52679 PMC4401378 25867716

[B91] Kleinewietfeld M. Manzel A. Titze J. Kvakan H. Yosef N. Linker R. A. (2013). Sodium chloride drives autoimmune disease by the induction of pathogenic TH17 cells. *Nature* 496 518–522. 10.1038/nature11868 23467095 PMC3746493

[B92] Koch-Henriksen N. Lauer K. (2017). Dietary sodium intake: An etiologic dead end in multiple sclerosis. *AAN Enterprises* 89, 1314–1315. 10.1212/WNL.0000000000004426 28842448

[B93] Koch-Henriksen N. Sørensen P. S. (2010). The changing demographic pattern of multiple sclerosis epidemiology. *Lancet Neurol.* 9 520–532. 20398859 10.1016/S1474-4422(10)70064-8

[B94] Koch-Henriksen N. Thygesen L. C. Stenager E. Laursen B. Magyari M. (2018). Incidence of MS has increased markedly over six decades in Denmark particularly with late onset and in women. *Neurology* 90:e1954–e1963. 10.1212/WNL.0000000000005612 29720546

[B95] Kouchaki E. Tamtaji O. R. Salami M. Bahmani F. Daneshvar Kakhaki R. Akbari E. (2017). Clinical and metabolic response to probiotic supplementation in patients with multiple sclerosis: A randomized, double-blind, placebo-controlled trial. *Clin. Nutr.* 36 1245–1249. 10.1016/j.clnu.2016.08.015 27669638

[B96] Krementsov D. N. Case L. K. Hickey W. F. Teuscher C. (2015). Exacerbation of autoimmune neuroinflammation by dietary sodium is genetically controlled and sex specific. *FASEB J.* 29 3446–3457. 10.1096/fj.15-272542 25917331 PMC4511199

[B97] Larsen J. P. Kvaale G. Riise T. Nyland H. Aarli J. A. (1984). An increase in the incidence of multiple sclerosis in western Norway. *Acta Neurol. Scand.* 70 96–103. 6333133 10.1111/j.1600-0404.1984.tb00809.x

[B98] Ledoux D. Labombardi V. J. Karter D. (2006). Lactobacillus acidophilus bacteraemia after use of a probiotic in a patient with AIDS and Hodgkin’s disease. *Int. J. Std Aids* 17 280–282. 10.1258/095646206776253507 16595054

[B99] Lee Y. K. Menezes J. S. Umesaki Y. Mazmanian S. K. (2011). Proinflammatory T-cell responses to gut microbiota promote experimental autoimmune encephalomyelitis. *Proc. Natl. Acad. Sci.U.S.A.* 108 4615–4622. 10.1073/pnas.1000082107 20660719 PMC3063590

[B100] Lherm T. Monet C. Nougière B. Soulier M. Larbi D. Le Gall C. (2002). Seven cases of fungemia with Saccharomyces boulardii in critically ill patients. *Intensive Care Med.* 28 797–801. 10.1007/s00134-002-1267-9 12107689

[B101] Li K. Wei S. Hu L. Yin X. Mai Y. Jiang C. (2020). Protection of Fecal Microbiota Transplantation in a Mouse Model of Multiple Sclerosis. *Mediat. Inflamm.* 2020:2058272. 10.1155/2020/2058272 32831634 PMC7426773

[B102] Liu Q. Yu Z. Tian F. Zhao J. Zhang H. Zhai Q. (2020). Surface components and metabolites of probiotics for regulation of intestinal epithelial barrier. *Microbial. Cell Fact.* 19:23. 10.1186/s12934-020-1289-4 32024520 PMC7003451

[B103] Liu S. Rezende R. M. Moreira T. G. Tankou S. K. Cox L. M. Wu M. (2019). Oral administration of miR-30d from feces of MS patients suppresses MS-like symptoms in mice by expanding Akkermansia muciniphila. *Cell Host Microbe* 26 779.–794. 10.1016/j.chom.2019.10.008 31784260 PMC6948921

[B104] Mackie R. I. Sghir A. Gaskins H. R. (1999). Developmental microbial ecology of the neonatal gastrointestinal tract. *Am. J. Clin. Nutr.* 69:1035S–1045S. 10232646 10.1093/ajcn/69.5.1035s

[B105] Makkawi S. Camara-Lemarroy C. Metz L. (2018). Fecal microbiota transplantation associated with 10 years of stability in a patient with SPMS. *Neurol. Neuroimmunol. Neuroinflamm.* 5:e459. 10.1212/NXI.0000000000000459 29619403 PMC5882466

[B106] Mancera P. Wappenhans B. Cordobilla B. Virgili N. Pugliese M. Rueda F. (2017). Natural docosahexaenoic acid in the triglyceride form attenuates *in vitro* microglial activation and ameliorates autoimmune encephalomyelitis in mice. *Nutrients* 9:681. 10.3390/nu9070681 28665331 PMC5537796

[B107] Mandić M. Mitić K. Nedeljković P. Perić M. Božić B. Lunić T. (2022). Vitamin B complex and experimental autoimmune encephalomyelitis &dash attenuation of the clinical signs and gut microbiota dysbiosis. *Nutrients* 14:1273. 35334928 10.3390/nu14061273 PMC8955508

[B108] Mangalam A. K. Murray J. (2019). Microbial monotherapy with Prevotella histicola for patients with multiple sclerosis. *Exp. Rev. Neurother.* 19 45–53. 10.1080/14737175.2019.1555473 30513004 PMC6548683

[B109] Mangalam A. Shahi S. K. Luckey D. Karau M. Marietta E. Luo N. (2017). Human gut-derived commensal bacteria suppress CNS inflammatory and demyelinating disease. *Cell Rep.* 20 1269–1277. 28793252 10.1016/j.celrep.2017.07.031 PMC5763484

[B110] Marrodan M. Alessandro L. Farez M. F. Correale J. (2019). The role of infections in multiple sclerosis. *Mult. Scler. J.* 25 891–901. 10.1177/1352458518823940 30638421

[B111] Martinez-Guryn K. Leone V. Chang E. B. (2019). Regional diversity of the gastrointestinal microbiome. *Cell Host Microbe* 26 314–324. 31513770 10.1016/j.chom.2019.08.011 PMC6750279

[B112] Massa J. O’Reilly E. Munger K. Ascherio A. (2013). Caffeine and alcohol intakes have no association with risk of multiple sclerosis. *Mult. Scler. J.* 19 53–58. 10.1177/1352458512448108 PMC3434262 22641303

[B113] Matsushima G. K. Morell P. (2001). The neurotoxicant, cuprizone, as a model to study demyelination and remyelination in the central nervous system. *Brain Pathol.* 11 107–116. 10.1111/j.1750-3639.2001.tb00385.x 11145196 PMC8098267

[B114] Matsushita T. Yanaba K. Bouaziz J.-D. Fujimoto M. Tedder T. F. (2008). Regulatory B cells inhibit EAE initiation in mice while other B cells promote disease progression. *J. Clin. Investig.* 118 3420–3430. 10.1172/JCI36030 18802481 PMC2542851

[B115] Mazdeh M. Mobaien A. R. (2012). Efficacy of doxycycline as add-on to interferon beta-1a in treatment of multiple sclerosis. *Iran. J. Neurol.* 11:70. 24250865 PMC3829238

[B116] McCarthy D. P. Richards M. H. Miller S. D. (2012). Mouse models of multiple sclerosis: Experimental autoimmune encephalomyelitis and Theiler’s virus-induced Demyelinating disease. *Methods Mol. Biol.* 900 381–401. 22933080 10.1007/978-1-60761-720-4_19 PMC3583382

[B117] McDonald J. Graves J. Waldman A. Lotze T. Schreiner T. Belman A. (2016). case-control study of dietary salt intake in pediatric-onset multiple sclerosis. *Mult. Scler. Relat. Disord.* 6 87–92. 27063630 10.1016/j.msard.2016.02.011 PMC4830915

[B118] McMurran C. E. Guzman de la Fuente A. Penalva R. Ben Menachem-Zidon O. Dombrowski Y. Falconer J. (2019). The microbiota regulates murine inflammatory responses to toxin-induced CNS demyelination but has minimal impact on remyelination. *Proc. Natl. Acad. Sci.U.S.A.* 116 25311–25321. 10.1073/pnas.1905787116 31740610 PMC6911206

[B119] McRae B. L. Kennedy M. K. Tan L.-J. Dal Canto M. C. Picha K. S. Miller S. D. (1992). Induction of active and adoptive relapsing experimental autoimmune encephalomyelitis (EAE) using an encephalitogenic epitope of proteolipid protein. *J. Neuroimmunol.* 38 229–240. 1376328 10.1016/0165-5728(92)90016-e

[B120] Melzer N. Meuth S. G. Torres-Salazar D. Bittner S. Zolzuya A. L. Weidenfeller C. (2008). A β-lactam antibiotic dampens excitotoxic inflammatory CNS damage in a mouse model of multiple sclerosis. *PLoS One* 3:e3149. 10.1371/journal.pone.0003149 18773080 PMC2522272

[B121] Mestas J. Hughes C. C. W. (2004). of mice and not men: Differences between mouse and human immunology. *J. Immunol.* 172 2731–2738. 14978070 10.4049/jimmunol.172.5.2731

[B122] Mestre L. Carrillo-Salinas F. J. Feliú A. Mecha M. Alonso G. Espejo C. (2020). How oral probiotics affect the severity of an experimental model of progressive multiple sclerosis? Bringing commensal bacteria into the neurodegenerative process. *Gut Microbes* 12:1813532. 10.1080/19490976.2020.1813532 32900255 PMC7524398

[B123] Mestre L. Carrillo-Salinas F. J. Mecha M. Mecha M. Feliú A. Espejo C. (2019). Manipulation of gut microbiota influences immune responses, axon preservation, and motor disability in a model of progressive multiple sclerosis. *Front. Immunol.* 10:1374. 10.3389/fimmu.2019.01374 31258540 PMC6587398

[B124] Metz L. M. Li D. K. Traboulsee A. L. Duquette P. Eliasziw M. Cerchiaro G. (2017). Trial of minocycline in a clinically isolated syndrome of multiple sclerosis. *N. Engl. J. Med.* 376 2122–2133. 28564557 10.1056/NEJMoa1608889

[B125] Millar J. H. Zilkha K. Langman M. Wright H. P. Smith A. D. Belin J. (1973). Double-blind trial of linoleate supplementation of the diet in multiple sclerosis. *Br. Med. J.* 1 765–768. 4571680 10.1136/bmj.1.5856.765 PMC1588925

[B126] Minagar A. Alexander J. S. Schwendimann R. N. Kelley R. E. Gonzalez-Toledo E. Jimenez J. J. (2008). Combination therapy with interferon beta-1a and doxycycline in multiple sclerosis: An open-label trial. *Arch. Neurol.* 65 199–204. 10.1001/archneurol.2007.41 18071030

[B127] Miranda P. M. De Palma G. Serkis V. Lu J. Louis-Auguste M. P. McCarville J. L. (2018). High salt diet exacerbates colitis in mice by decreasing *Lactobacillus* levels and butyrate production. *Microbiome* 6:57. 10.1186/s40168-018-0433-4 29566748 PMC5865374

[B128] Miyake S. Kim S. Suda W. Oshima K. Nakamura M. Matsuoka T. (2015). Dysbiosis in the gut microbiota of patients with multiple sclerosis, with a striking depletion of species belonging to clostridia XIVa and IV clusters. *PLoS One* 10:e0137429. 10.1371/journal.pone.0137429 26367776 PMC4569432

[B129] Miyauchi E. Kim S. W. Suda W. Kawasumi M. Onawa S. Taguchi-Atarashi N. (2020). Gut microorganisms act together to exacerbate inflammation in spinal cords. *Nature* 585 102–106. 10.1038/s41586-020-2634-9 32848245

[B130] Miyauchi E. Shimokawa C. Steimle A. Desai M. S. Ohno H. (2022). The impact of the gut microbiome on extra-intestinal Autoimmune diseases. *Nat. Rev. Immunol.* [Epub ahead of print]. 10.1038/s41577-022-00727-y 35534624

[B131] Mizuno M. Noto D. Kaga N. Chiba A. Miyake S. (2017). The dual role of short fatty acid chains in the pathogenesis of autoimmune disease models. *PLoS One* 12:e0173032. 10.1371/journal.pone.0173032 28235016 PMC5325617

[B132] Montgomery T. L. Kunstner A. Kennedy J. J. Fang Q. Asarian L. Culp-Hill R. (2020). Interactions between host genetics and gut microbiota determine susceptibility to CNS autoimmunity. *Proc. Natl. Acad. Sci. U.S.A.* 117 27516–27527. 10.1073/pnas.2002817117 33077601 PMC7959502

[B133] Moritz M. L. Ayus J. C. (2010). Water water everywhere: Standardizing postoperative fluid therapy with 0.9% normal saline. *Anesth. Analg.* 110 293–295. 10.1213/ANE.0b013e3181c98131 20081126

[B134] Na S. Y. Janakiraman M. Leliavski A. Krishnamoorthy G. (2021). High-salt diet suppresses autoimmune demyelination by regulating the blood-brain barrier permeability. *Proc. Natl. Acad. Sci. U.S.A.* 118:e2025944118. 10.1073/pnas.2025944118 33723078 PMC7999868

[B135] Nair A. B. Jacob S. A. (2016). A simple practice guide for dose conversion between animals and human. *J. Basic Clin. Pharmacy* 7:27–31. 10.4103/0976-0105.177703 27057123 PMC4804402

[B136] Navarro-López V. Méndez-Miralles M. Á Vela-Yebra R. Fríes-Ramos A. Sánchez-Pellicer P. Ruzafa-Costas B. (2022). Gut microbiota as a potential predictive biomarker in relapsing-remitting multiple sclerosis. *Genes* 13:930. 10.3390/genes13050930 35627315 PMC9140870

[B137] Nijeholt G. Van Walderveen M. Castelijns J. van Waesberghe J. H. Polman C. Scheltens P. (1998). Brain and spinal cord abnormalities in multiple sclerosis. Correlation between MRI parameters, clinical subtypes and symptoms. *Brain* 121 687–697. 9577394 10.1093/brain/121.4.687

[B138] Nørgaard M. Nielsen R. B. Jacobsen J. B. Gradus J. L. Stenager E. Koch-Henriksen N. (2011). Use of penicillin and other antibiotics and risk of multiple sclerosis: A population-based case-control study. *Am. J. Epidemiol.* 174 945–948. 21920946 10.1093/aje/kwr201

[B139] Nourbakhsh B. Graves J. Casper T. C. Lulu S. Waldman A. Belman A. (2016). Dietary salt intake and time to relapse in paediatric multiple sclerosis. *J. Neurol. Neurosurg. Psychiatry* 87 1350–1353. 27343226 10.1136/jnnp-2016-313410 PMC5370574

[B140] Ochoa-Repáraz J. Kasper L. H. (2017). The influence of gut-derived CD39 regulatory T cells in CNS Demyelinating disease. *Transl. Res.* 179 126–138. 10.1016/j.trsl.2016.07.016 27519147 PMC5164971

[B141] Ochoa-Repáraz J. Kirby T. O. Kasper L. H. (2018). The gut microbiome and multiple sclerosis. *Cold Spring Harb. Perspect Med.* 8:a029017. 29311123 10.1101/cshperspect.a029017 PMC5983160

[B142] Ochoa-Reparaz J. Mielcarz D. W. Ditrio L. E. Burroughs A. R. Foureau D. M. Haque-Begum S. (2009). Role of gut commensal microflora in the development of experimental autoimmune encephalomyelitis. *J. Immunol.* 183 6041–6050. 19841183 10.4049/jimmunol.0900747

[B143] Ochoa-Repáraz J. Mielcarz D. W. Haque-Begum S. Kasper L. H. (2010a). Induction of a regulatory B cell population in experimental allergic encephalomyelitis by alteration of the gut commensal microflora. *Gut Microbes* 1 103–108. 10.4161/gmic.1.2.11515 21326918 PMC3023588

[B144] Ochoa-Repáraz J. Mielcarz D. Wang Y. Begum-Haque S. Dasgupta S. Kasper D. L. (2010b). A polysaccharide from the human commensal *Bacteroides* fragilis protects against CNS demyelinating disease. *Mucosal. Immunol.* 3 487–495. 10.1038/mi.2010.29 20531465

[B145] Oggioni M. R. Pozzi G. Valensin P. E. Galieni P. Bigazzi C. (1998). Recurrent septicemia in an immunocompromised patient due to probiotic strains of Bacillus subtilis. *J. Clin. Microbiol.* 36 325–326. 10.1128/JCM.36.1.325-326.1998 9431982 PMC124869

[B146] Ohland C. L. MacNaughton W. K. (2010). Probiotic bacteria and intestinal epithelial barrier function. *Am. J. Physiol. Gastrointest. Liver Physiol.* 298:G807–G819. 20299599 10.1152/ajpgi.00243.2009

[B147] Opazo M. C. Ortega-Rocha E. M. Coronado-Arrázola I. Bonifaz L. C. Boudin H. Neunlist M. (2018). Intestinal microbiota influences non-intestinal related Autoimmune diseases. *Front. Microbiol.* 9:432. 10.3389/fmicb.2018.00432 29593681 PMC5857604

[B148] Pakpoor J. Goldacre R. Disanto G. Giovannoni G. Goldacre M. J. (2014). Alcohol misuse disorders and multiple sclerosis risk. *JAMA Neurol.* 71 1188–1189. 25200540 10.1001/jamaneurol.2014.1795

[B149] Park J. Wang Q. Wu Q. Mao-Draayer Y. Kim C. H. (2019). Bidirectional regulatory potentials of short-chain fatty acids and their G-protein-coupled receptors in autoimmune neuroinflammation. *Sci. Rep.* 9:8837. 31222050 10.1038/s41598-019-45311-y PMC6586800

[B150] Parker A. Fonseca S. Carding S. R. (2020). Gut microbes and metabolites as modulators of blood-brain barrier integrity and brain health. *Gut Microbes* 11 135–157. 10.1080/19490976.2019.1638722 31368397 PMC7053956

[B151] Pérez-Pérez S. Domínguez-Mozo M. I. Alonso-Gómez A. Medina S. Villarrubia N. Fernández-Velasco J. I. (2020). Acetate correlates with disability and immune response in multiple sclerosis. *PeerJ* 8:e10220. 10.7717/peerj.10220 33240608 PMC7676361

[B152] Pfortmueller C. A. Schefold J. C. (2017). Hypertonic saline in critical illness-a systematic review. *J. Crit. Care* 42 168–177. 28746899 10.1016/j.jcrc.2017.06.019

[B153] Pröbstel A. K. Zhou X. Baumann R. Wischnewski S. Kutza M. Rojas O. L. (2020). Gut microbiota-specific IgA(+) B cells traffic to the CNS in active multiple sclerosis. *Sci. Immunol.* 5:eabc7191. 10.1126/sciimmunol.abc7191 33219152 PMC8043673

[B154] Pruett S. Tan W. Howell I. I. I. G. E. Nanduri B. (2020). Dosage scaling of alcohol in binge exposure models in mice: An empirical assessment of the relationship between dose, alcohol exposure, and peak blood concentrations in humans and mice. *Alcohol* 89 9–17. 10.1016/j.alcohol.2020.03.011 32259574 PMC8221372

[B155] Qin J. Li R. Raes J. Arumugam M. Burgdorf K. S. Manichanh C. (2010). human gut microbial gene catalogue established by metagenomic sequencing. *Nature* 464 59–65. 20203603 10.1038/nature08821 PMC3779803

[B156] Quevrain E. Maubert M. A. Michon C. Chain F. Marquant R. Tailhades J. (2016). Identification of an anti-inflammatory protein from Faecalibacterium prausnitzii, a commensal bacterium deficient in Crohn’s disease. *Gut* 65 415–425. 26045134 10.1136/gutjnl-2014-307649 PMC5136800

[B157] Quévrain E. Maubert M. Michon C. Chain F. Marquant R. Tailhades J. (2016). Identification of an anti-inflammatory protein from Faecalibacterium prausnitzii, a commensal bacterium deficient in Crohn’s disease. *Gut* 65 415–425. 10.1136/gutjnl-2014-307649 26045134 PMC5136800

[B158] Quraishi M. N. Widlak M. Bhala Na Moore D. Price M. Sharma N. (2017). Systematic review with meta-analysis: The efficacy of faecal microbiota transplantation for the treatment of recurrent and refractory Clostridium difficile infection. *Aliment. Pharmacol. Ther.* 46 479–493. 28707337 10.1111/apt.14201

[B159] Rahimlou M. Hosseini S. A. Majdinasab N. Haghighizadeh M. H. Husain D. (2022). Effects of long-term administration of Multi-Strain Probiotic on circulating levels of BDNF, NGF, IL-6 and mental health in patients with multiple sclerosis: A randomized, double-blind, placebo-controlled trial. *Nutr. Neurosci.* 25 411–422. 10.1080/1028415X.2020.1758887 32500827

[B160] Ramirez-Ramirez V. Macias-Islas M. A. Ortiz G. G. Pacheco-Moises F. Torres-Sanchez, Sorto-Gomez T. E. (2013). Efficacy of fish oil on serum of TNF*A*, IL-1β, and IL-6 oxidative stress markers in multiple sclerosis treated with interferon beta-1b. *Oxid. Med. Cell. Longev.* 2013:709493. 10.1155/2013/709493 23861993 PMC3703725

[B161] Ransohoff R. M. (2012). Animal models of multiple sclerosis: The good, the bad and the bottom line. *Nat. Neurosci.* 15 1074–1077. 10.1038/nn.3168 22837037 PMC7097342

[B162] Rao K. Safdar N. (2016). Fecal microbiota transplantation for the treatment of Clostridium difficile infection. *J. Hosp. Med.* 11 56–61. 26344412 10.1002/jhm.2449 PMC4908581

[B163] Ropper A. H. (2012). Hyperosmolar therapy for raised intracranial pressure. *N. Engl. J. Med.* 367 746–752. 22913684 10.1056/NEJMct1206321

[B164] Rudick R. Polman C. Clifford D. Miller D. Steinman L. (2013). Natalizumab: Bench to Bedside and Beyond. *JAMA Neurol.* 70 172–182. 10.1001/jamaneurol.2013.598 23128399

[B165] Salami M. Kouchaki E. Asemi Z. Tamtaji O. R. (2019). How probiotic bacteria influence the motor and mental behaviors as well as immunological and oxidative biomarkers in multiple sclerosis? A double blind clinical trial. *J. Funct. Foods* 52 8–13.

[B166] Salehipour Z. Haghmorad D. Sankian M. Rastin M. Nosratabadi R. Soltan Dallal M. M. (2017). Bifidobacterium animalis in combination with human origin of Lactobacillus plantarum ameliorate neuroinflammation in experimental model of multiple sclerosis by altering CD4+ T cell subset balance. *Biomed. Pharmacother.* 95 1535–1548. 10.1016/j.biopha.2017.08.117 28946394

[B167] Salvati S. Natali F. Attorri L. Benedetto R. Leonardi F. Di Biase A. (2008). Eicosapentaenoic acid stimulates the expression of myelin proteins in rat brain. *J. Neurosci. Res.* 86 776–784. 17941053 10.1002/jnr.21537

[B168] Salvati S. Natali F. Attorri L. Raggi C. Biase A. D. Sanchez M. (2004). Stimulation of myelin proteolipid protein gene expression by eicosapentaenoic acid in C6 glioma cells. *Neurochem. Int.* 44 331–338. 14643750 10.1016/s0197-0186(03)00172-4

[B169] Saresella M. Marventano I. Barone M. La Rosa F. Piancone F. Mendozzi L. (2020). Alterations in circulating fatty acid are associated with gut microbiota dysbiosis and inflammation in multiple sclerosis. *Front. Immunol.* 11:1390. 10.3389/fimmu.2020.01390 32733460 PMC7358580

[B170] Schilling S. Goelz S. Linker R. Luehder F. Gold R. (2006). Fumaric acid esters are effective in chronic experimental autoimmune encephalomyelitis and suppress macrophage infiltration. *Clin. Exp. Immunol.* 145 101–107. 10.1111/j.1365-2249.2006.03094.x 16792679 PMC1942010

[B171] Schumann J. Leichtle A. Thiery J. Fuhrmann H. (2011). Fatty acid and peptide profiles in plasma membrane and membrane rafts of PUFA supplemented RAW264. 7 macrophages. *PLoS One* 6:e24066. 10.1371/journal.pone.0024066 21887374 PMC3161109

[B172] Scrivo R. Massaro L. Barbati C. Vomero M. Ceccarelli F. Spinelli F. R. (2017). The role of dietary sodium intake on the modulation of T helper 17 cells and regulatory T cells in patients with rheumatoid arthritis and systemic lupus erythematosus. *PLoS One* 12:e0184449. 10.1371/journal.pone.0184449 28877244 PMC5587319

[B173] Seifert H. A. Benedek G. Nguyen H. Gerstner G. Zhang Y. Kent G. (2018). Antibiotics Protect against EAE by increasing regulatory and anti-inflammatory cells. *Metab. Brain Dis*. 33, 1599–1607. 10.1007/s11011-018-0266-7 29916184 PMC6298859

[B174] Shahi S. K. Freedman S. N. Murra A. C. Zarei K. Sompallae R. Gibson-Corley K. N. (2019). Prevotella histicola, a human gut commensal, is as potent as COPAXONE^®^ in an animal model of multiple sclerosis. *Front. Immunol.* 10:462. 10.3389/fimmu.2019.00462 30984162 PMC6448018

[B175] Silva Y. P. Bernardi A. Frozza R. L. (2020). The role of short-chain fatty acids from gut microbiota in gut-brain communication. *Front. Endocrinol.* 11:25. 10.3389/fendo.2020.00025 32082260 PMC7005631

[B176] Simmons S. B. Pierson E. R. Lee S. Y. Goverman J. M. (2013). Modeling the heterogeneity of multiple sclerosis in animals. *Trends Immunol.* 34 410–422. 23707039 10.1016/j.it.2013.04.006 PMC3752929

[B177] Singh S. Khanna D. Kalra S. (2021). Minocycline and doxycycline: More than antibiotics. *Curr. Mol. Pharmacol.* 14 1046–1065. 33568043 10.2174/1874467214666210210122628

[B178] Smith K. J. McDonald W. (1999). The pathophysiology of multiple sclerosis:: the mechanisms underlying the production of symptoms and the natural history of the disease. *Philos. Trans. R. Soc. Lond. Series B* 354 1649–1673. 10.1098/rstb.1999.0510 10603618 PMC1692682

[B179] Smith P. A. Heijmans N. Ouwerling B. Breij E. C. Evans N. van Noort J. M. (2005). Native myelin oligodendrocyte glycoprotein promotes severe chronic neurological disease and demyelination in Biozzi ABH mice. *Eur. J. Immunol.* 35 1311–1319. 10.1002/eji.200425842 15761848

[B180] Sokovic-Bajic S. Mihajlovic S. Radojevic D. Popović D. Djokic J. Stanisavljević S. (2020). *J. Serb. Chem. Soc.* 85 163–176.

[B181] Spichak S. Donoso F. Moloney G. M. Gunnigle E. Brown J. M. Codagnone M. (2021). Microbially-derived short-chain fatty acids impact astrocyte gene expression in a sex-specific manner. *Brain Behav. Immun. Health* 16:100318. 10.1016/j.bbih.2021.100318 34589808 PMC8474187

[B182] Stanisavljević S. Čepić A. Bojić S. Veljović K. Mihajlović S. Ðedović N. (2019). Oral neonatal antibiotic treatment perturbs gut microbiota and aggravates central nervous system autoimmunity in Dark Agouti rats. *Sci. Rep.* 9:918. 10.1038/s41598-018-37505-7 30696913 PMC6351648

[B183] Stavropoulos F. Georgiou E. Sargiannidou I. Kleopa K. A. (2021). Dysregulation of blood-brain barrier and exacerbated inflammatory response in Cx47-deficient mice after induction of EAE. *Pharmaceuticals* 14:621. 10.3390/ph14070621 34203192 PMC8308522

[B184] Steinman L. (2005). Blocking adhesion molecules as therapy for multiple sclerosis: Natalizumab. *Nat. Rev. Drug Discov.* 4 510–518. 10.1038/nrd1752 15931259

[B185] Strzępa A. Lobo F. M. Majewska-Szczepanik M. Szczepanik M. (2018). Antibiotics and autoimmune and allergy diseases: Causative factor or treatment? *Int. Immunopharmacol.* 65 328–341. 30359934 10.1016/j.intimp.2018.10.021

[B186] Sundström B. Johansson I. Rantapää-Dahlqvist S. (2015). Interaction between dietary sodium and smoking increases the risk for rheumatoid arthritis: Results from a nested case–control study. *Rheumatology* 54 487–493. 10.1093/rheumatology/keu330 25209067

[B187] Svenningsson A. Runmarker B. Lycke J. Andersen O. (1990). Incidence of MS during two fifteen-year periods in the Gothenburg region of Sweden. *Acta Neurol. Scand.* 82 161–168. 10.1111/j.1600-0404.1990.tb04483.x 2270743

[B188] Swank R. L. (1950). Multiple sclerosis: A correlation of its incidence with dietary fat. *Am. J. Med. Sci.* 220 421–430. 10.1097/00000441-195022040-00011 14771073

[B189] Swank R. L. Goodwin J. (2003). Review of MS patient survival on a Swank low saturated fat diet 1 1 (For an additional perspective, see editorial opinions). *Nutrition* 19:161. 10.1016/S0899-9007(02)00851-1 12591551

[B190] Swank R. L. Lerstad O. Strøm A. Backer J. (1952). Multiple sclerosis in rural Norway: Its geographic and occupational incidence in relation to nutrition. *N. Engl. J. Med.* 246 721–728. 10.1056/NEJM195205082461901 14929306

[B191] Swidsinski A. Dörffel Y. Loening-Baucke V. Gille C. Göktas Ö Reißhauer A. (2017). Reduced mass and diversity of the colonic microbiome in patients with multiple sclerosis and their improvement with ketogenic diet. *Front. Microbiol.* 8:1141. 10.3389/fmicb.2017.01141 28702003 PMC5488402

[B192] Takewaki D. Suda W. Sato W. Takayasu L. Kumar N. Kimura K. (2020). Alterations of the gut ecological and functional microenvironment in different stages of multiple sclerosis. *Proc. Natl. Acad. Sci. U.S.A.* 117 22402–22412. 10.1073/pnas.2011703117 32839304 PMC7486801

[B193] Teitelbaum D. Fridkis-Hareli M. Arnon R. Sela M. (1996). Copolymer 1 inhibits chronic relapsing experimental allergic encephalomyelitis induced by proteolipid protein (PLP) peptides in mice and interferes with PLP-specific T cell responses. *J. Neuroimmunol.* 64 209–217. 10.1016/0165-5728(95)00180-8 8632064

[B194] Torkildsen Ø Brunborg L. A. Milde A. M. Mørk S. J. Myhr K.-M. Bø L. (2009). A salmon based diet protects mice from behavioural changes in the cuprizone model for demyelination. *Clin. Nutr.* 28 83–87. 10.1016/j.clnu.2008.10.015 19042061

[B195] Torkildsen Ø Wergeland S. Bakke S. Beiske A. G. Bjerve K. S. Hovdal H. (2012). ω-3 Fatty Acid Treatment in Multiple Sclerosis (OFAMS Study): A Randomized, Double-Blind, Placebo-Controlled Trial. *Arch. Neurol.* 69 1044–1051. 10.1001/archneurol.2012.283 22507886

[B196] Tremlett H. Fadrosh D. W. Faruqi A. A. Zhu F. Hart J. Roalstad S. (2016). Gut microbiota in early pediatric multiple sclerosis: A case–control study. *Eur. J. Neurol.* 23 1308–1321. 10.1111/ene.13026 27176462 PMC4955679

[B197] Trend S. Leffler J. Jones A. P. Cha L. Gorman S. Brown D. A. (2021). Associations of serum short-chain fatty acids with circulating immune cells and serum biomarkers in patients with multiple sclerosis. *Sci. Rep.* 11:5244. 33664396 10.1038/s41598-021-84881-8 PMC7933417

[B198] Tsai H.-C. Huang Y. Garris C. S. Moreno M. A. Griffin C. W. Han M. H. (2016). Effects of sphingosine-1-phosphate receptor 1 phosphorylation in response to FTY720 during neuroinflammation. *JCI Insight* 1:e86462. 27699272 10.1172/jci.insight.86462 PMC5033897

[B199] Unoda K. Doi Y. Nakajima H. Yamane K. Hosokawa T. Ishida S. (2013). Eicosapentaenoic acid (EPA) induces peroxisome proliferator-activated receptors and ameliorates experimental autoimmune encephalomyelitis. *J. Neuroimmunol.* 256 7–12. 10.1016/j.jneuroim.2012.12.003 23276800

[B200] van den Berg E. Geleijnse J. M. Brink E. J. van Baak M. A. Homan van der Heide J. J. Gans R. O. (2012). Sodium intake and blood pressure in renal transplant recipients. *Nephrol. Dial. Transplant.* 27 3352–3359. 10.1093/ndt/gfs069 22499024

[B201] Ventura R. Iizumi T. Battaglia T. Liu M. Perez-Perez G. I. Herbert J. (2019). Gut microbiome of treatment-naïve MS patients of different ethnicities early in disease course. *Sci. Rep.* 9:16396. 10.1038/s41598-019-52894-z 31705027 PMC6841666

[B202] Wallin M. T. Culpepper W. J. Campbell J. D. Nelson L. M. Langer-Gould A. Marrie R. A. (2019). The prevalence of MS in the United States: A population-based estimate using health claims data. *Neurology* 92:e1029–e1040. 30770430 10.1212/WNL.0000000000007035 PMC6442006

[B203] Walton C. King R. Rechtman L. Kaye W. Leray E. Marrie R. A. (2020). Rising prevalence of multiple sclerosis worldwide: Insights from the Atlas of MS. *Mult. Scler. J.* 26 1816–1821. 10.1177/1352458520970841 33174475 PMC7720355

[B204] Wang D. Lu Z. Hu L. Zhang Y. Hu X. (2009). Macrolide antibiotics aggravate experimental autoimmune encephalomyelitis and inhibit inducible nitric oxide synthase. *Immunol. Invest.* 38, 602–612. 10.1080/08820130903062194 19811424

[B205] Wang S. Chen H. Wen X. Mu J. Sun M. Song X. (2021). The Efficacy of Fecal Microbiota Transplantation in Experimental Autoimmune Encephalomyelitis: Transcriptome and Gut Microbiota Profiling. *J. Immunol. Res.* 2021:4400428. 10.1155/2021/4400428 34938813 PMC8687821

[B206] Wang X. Wang B.-R. Zhang X.-J. Xu Z. Ding Y.-Q. Ju G. (2002). Evidences for vagus nerve in maintenance of immune balance and transmission of immune information from gut to brain in STM-infected rats. *World J. Gastroenterol.* 8:540. 10.3748/wjg.v8.i3.540 12046088 PMC4656439

[B207] Weber M. S. Prod’Homme T. Patarroyo J. C. Molnarfi N. Karnezis T. Lehmann-Horn K. (2010). B-cell activation influences T-cell polarization and outcome of anti-CD20 B-cell depletion in central nervous system autoimmunity. *Ann. Neurol.* 68 369–383. 10.1002/ana.22081 20641064 PMC3375897

[B208] Weiner H. L. Mackin G. A. Matsui M. Orav E. J. Khoury S. J. Dawson D. M. (1993). Double-blind pilot trial of oral tolerization with myelin antigens in multiple sclerosis. *Science* 259 1321–1324. 10.1126/science.7680493 7680493

[B209] Weinstock-Guttman B. Baier M. Park Y. Feichter J. Lee-Kwen P. Gallagher E. (2005). Low fat dietary intervention with ω-3 fatty acid supplementation in multiple sclerosis patients. *Prostaglandins Leukot. Essent. Fatty Acids* 73 397–404. 10.1016/j.plefa.2005.05.024 16099630

[B210] Wilck N. Matus M. G. Kearney S. M. Olesen S. W. Forslund K. Bartolomaeus H. (2017). Salt-responsive gut commensal modulates TH17 axis and disease. *Nature* 551 585–589. 10.1038/nature24628 29143823 PMC6070150

[B211] Wu B. Yang D. Yang S. Zhang G. (2021). Dietary salt intake and gastric cancer risk: A systematic review and meta-analysis. *Front. Nutr.* 8:801228. 10.3389/fnut.2021.801228 34957192 PMC8692376

[B212] Wu C. Yosef N. Thalhamer T. Zhu C. Xiao S. Kishi Y. (2013). Induction of pathogenic TH17 cells by inducible salt-sensing kinase SGK1. *Nature* 496 513–517. 10.1038/nature11984 23467085 PMC3637879

[B213] Yednock T. A. Cannon C. Fritz L. C. Sanchez-Madrid F. Steinman L. Karin N. (1992). Prevention of experimental autoimmune encephalomyelitis by antibodies against alpha 4 beta 1 integrin. *Nature* 356 63–66. 1538783 10.1038/356063a0

[B214] Yokote H. Miyake S. Croxford J. L. Oki S. Mizusawa H. Yamamura T. N. K. T. (2008). NKT cell-dependent amelioration of a mouse model of multiple sclerosis by altering gut flora. *Am. J. Pathol.* 173 1714–1723. 10.2353/ajpath.2008.080622 18974295 PMC2626383

[B215] Zarghami A. Li Y. Claflin S. B. van der Mei I. Taylor B. V. (2021). Role of environmental factors in multiple sclerosis. *Expert Rev. Neurother.* 21 1389–1408. 34494502 10.1080/14737175.2021.1978843

[B216] Zeng Q. Junli G. Liu X. Chen C. Sun X. Li H. (2019). Gut dysbiosis and lack of short chain fatty acids in a Chinese cohort of patients with multiple sclerosis. *Neurochem. Int.* 129:104468. 10.1016/j.neuint.2019.104468 31108132

[B217] Zeraati M. Enayati M. Kafami L. Shahidi S. H. Salari A.-A. (2019). Gut microbiota depletion from early adolescence alters adult immunological and neurobehavioral responses in a mouse model of multiple sclerosis. *Neuropharmacology* 157:107685. 10.1016/j.neuropharm.2019.107685 31247271

[B218] Zheng J. Wittouck S. Salvetti E. Franz C. M. A. P. Harris H. M. B. Mattarelli P. (2020). taxonomic note on the genus Lactobacillus: Description of 23 novel genera, emended description of the genus Lactobacillus Beijerinck 1901, and union of Lactobacillaceae and Leuconostocaceae. *Int. J. Syst. Evol. Microbiol.* 70 2782–2858. 10.1099/ijsem.0.004107 32293557

[B219] Zhou X. Baumann R. Gao X. (2022). Gut microbiome of multiple sclerosis patients and paired household healthy controls reveal associations with disease risk and course. *Cell* 185 3467–3486.e16. 10.1016/j.cell.2022.08.021 36113426 PMC10143502

